# 2011 SOSORT guidelines: Orthopaedic and Rehabilitation treatment of idiopathic scoliosis during growth

**DOI:** 10.1186/1748-7161-7-3

**Published:** 2012-01-20

**Authors:** Stefano Negrini, Angelo G Aulisa, Lorenzo Aulisa, Alin B Circo, Jean Claude de Mauroy, Jacek Durmala, Theodoros B Grivas, Patrick Knott, Tomasz Kotwicki, Toru Maruyama, Silvia Minozzi, Joseph P O'Brien, Dimitris Papadopoulos, Manuel Rigo, Charles H Rivard, Michele Romano, James H Wynne, Monica Villagrasa, Hans-Rudolf Weiss, Fabio Zaina

**Affiliations:** 1 Physical and Rehabilitation Medicine, University of Brescia, Italy; 2 Don Gnocchi Foundation, Milan, Italy; 3 ISICO (Italian Scientific Spine Institute), Milan, Italy; 4 Orthopaedics and Traumatology Division, Bambino Gesù Children's Hospital, Institute of Scientific Research, Piazza San Onofrio 4, 00165, Rome, Italy; 5 Department of Orthopaedics, Catholic University of the Sacred Heart, University Hospital 'Agostino Gemelli', L.go F. Vito, 1-00168 Rome, Italy; 6 Sainte Justine Hospital, University of Montreal, Canada; 7 Clinique du Parc, Lyon, France; 8 Department of Rehabilitation, Medical University of Silesia and University Hospital, Katowice, Poland; 9 Department of Trauma and Orthopaedics, "Tzanio" General Hospital, Tzani and Afendouli 1 st, Piraeus 18536, Greece; 10 Rosalind Franklin University of Medicine and Science, North Chicago, Illinois, 60064, USA; 11 Spine Disorders Unit, Department of Pediatric Orthopedics and Traumatology, University of Medical Sciences, Poznan, Poland; 12 Department of Orthopaedic Surgery, Saitama Medical Center, Saitama Medical University, Japan; 13 Cochrane Review Group on Drugs and Alcohol. Department of Epidemiology. Lazio Region. Via di Santa Costanza, 53. 00198 Rome. Italy; 14 National Scoliosis Foundation, Boston, USA; 15 Spondylos Laser Spine Lab, Orthopaedic Facility and Rehabilitation Center, 74, Messogion Ave, 115 27, Athens, Greece; 16 Institut Elena Salvá. Vía Augusta 185. 08021 Barcelona, Spain; 17 Boston Brace Co., Boston, USA; 18 Gesundheitsforum Nahetal. Alzeyer Str. 23. D-55457 Gensingen, Germany

## Abstract

**Background:**

The International Scientific Society on Scoliosis Orthopaedic and Rehabilitation Treatment (SOSORT), that produced its first Guidelines in 2005, felt the need to revise them and increase their scientific quality. The aim is to offer to all professionals and their patients an evidence-based updated review of the actual evidence on conservative treatment of idiopathic scoliosis (CTIS).

**Methods:**

All types of professionals (specialty physicians, and allied health professionals) engaged in CTIS have been involved together with a methodologist and a patient representative. A review of all the relevant literature and of the existing Guidelines have been performed. Documents, recommendations, and practical approach flow charts have been developed according to a Delphi procedure. A methodological and practical review has been made, and a final Consensus Session was held during the 2011 Barcelona SOSORT Meeting.

**Results:**

The contents of the document are: methodology; generalities on idiopathic scoliosis; approach to CTIS in different patients, with practical flow-charts; literature review and recommendations on assessment, bracing, physiotherapy, Physiotherapeutic Specific Exercises (PSE) and other CTIS. Sixty-five recommendations have been given, divided in the following topics: Bracing (20 recommendations), PSE to prevent scoliosis progression during growth (8), PSE during brace treatment and surgical therapy (5), Other conservative treatments (3), Respiratory function and exercises (3), Sports activities (6), Assessment (20). No recommendations reached a Strength of Evidence level I; 2 were level II; 7 level III; and 20 level IV; through the Consensus procedure 26 reached level V and 10 level VI. The Strength of Recommendations was Grade A for 13, B for 49 and C for 3; none had grade D.

**Conclusion:**

These Guidelines have been a big effort of SOSORT to paint the actual situation of CTIS, starting from the evidence, and filling all the gray areas using a scientific method. According to results, it is possible to understand the lack of research in general on CTIS. SOSORT invites researchers to join, and clinicians to develop good research strategies to allow in the future to support or refute these recommendations according to new and stronger evidence.

## Premise

### Mandate

The international Scientific Society on Scoliosis Orthopaedic and Rehabilitation Treatment (SOSORT), that produced its first Guidelines during the 2005 Milan Meeting, and published them in 2006 in the Journal Scoliosis [1], felt the need to revise them and increase their scientific quality. During the SOSORT 2010 Meeting in Montreal the SOSORT Guidelines Commission was established, coordinated by Stefano Negrini. The Mandate to the Commission was to develop Guidelines methodologically sound and evidence based, giving recommendations according to the strength of the actual evidence.

### Commission

The Commission was open to all SOSORT Members who decided to adhere to the project; it has been decided to include also a methodologist (Silvia Minozzi), while a patient (Joe P O'Brien), member of SOSORT and President of the US National Scoliosis Foundation, has been nominated as an external judge with the patients' perspective.

### Content

The contents of the document of the 2011 SOSORT Guidelines on "Orthopaedic and Rehabilitation Treatment of Idiopathic Scoliosis During Growth" are:

1. Methodology

2. Generalities on idiopathic scoliosis

3. Approach to conservative treatment of idiopathic scoliosis in different patients, with practical flow-charts

4. Literature review and recommendations on assessment, bracing, physiotherapy, Physiotherapeutic Specific Exercises and other conservative treatments

An Appendix (Additional File 1) has been added to give all details the Method used to develop the Guidelines.

### Scope, purpose and applications

The aim of these Guidelines is to offer to all professionals engaged in the conservative treatment of scoliosis an evidence-based updated review of the actual evidence in the field, together with a series of evidence-based recommendations. The multiple gray areas, important for the every day clinical practice, in which it is not possible to give an evidence-based recommendation, have been covered through a formal and explicit consensus methodology, as outlined in the Appendix (Additional File 1), to provide a consensus recommendation.

The Guidelines are meant to apply to all idiopathic scoliosis patients regardless of age. The main clinical questions that they cover are:

• Which assessment of the patient should be performed?

• Which conservative treatment should be provided, and how?

• How and when should bracing be applied?

• How and when should exercises be used?

### Development of the Guidelines

All types of professionals engaged in the conservative treatment of scoliosis have been involved: specialty physicians (orthopaedics, physical and rehabilitation medicine, psychiatry...) and allied health professionals (orthotists, physiotherapists, chiropractors...); a methodologist and a patient representative have been included as well.

Nevertheless, it must be underlined that these Guidelines have been developed by the SOSORT, that is the Society on Scoliosis treatment that is focused exclusively in the conservative approach to scoliosis. The other two international Scientific Societies involved in scoliosis treatment, while considering also the conservative approach, focus mainly either in the surgical treatment (Scoliosis Research Society) or in general research (International Research Society on Spinal Deformities): the SRS and IRSSD have not been involved in this Guidelines development, even if members of these Societies are also members of the SOSORT and participated.

Patients have been involved in the development of the Guidelines through the US National Scoliosis Foundation, representing 25,000 actual scoliosis patients.

### Methods

Methods are outlined in all details in the Appendix (Additional File 1).

For the treatment sections we performed systematic reviews of the literature in February 2011. Medline was searched from its inception, with no language limitations. The search strategies, the selection criteria, and the number of retrieved papers are listed in the individual sections. We also searched: the abstracts of all SOSORT Meetings, from the first one in 2003 to 2010; the personal files and knowledge of all the authors; the papers retrieved with all the other searches listed in these Guidelines; the references sections of all retrieved papers.

To produce the actual Guidelines, a review of the previous ones has been performed: these have been searched through a comprehensive bibliographic search on Medline with the key word "Scoliosis" and "Guidelines" [1-4]. The final documents, recommendations, and practical approach flow charts have been developed according to a Delphi procedure carefully listed in the Appendix (Additional File 1). A methodological and practical review have been made, and a final Consensus Session held during the 2011 Barcelona SOSORT Meeting.

A classical Strength of Evidence (SoE) table has been adopted (Table 1). According to the Italian Guidelines [2], levels V and VI have been added according to the Consensus session held during the SOSORT Meeting. A Strength of Recommendation (SoR) scale has also been used (Table 2), that assumes that each Recommendation should have in the clinical everyday world, balancing all typical factors involved in this decision (patients, professionals, social). The SoR scale is meant to accompany and complement the Strength of Evidence scale.

**Table 1:** Strength of Evidence grading used in these Guidelines.

Strength of evidence	Question	Meaning
**I**	*Effectiveness*	Multiple Randomized Controlled Trials or Systematic Reviews of such studies
	
	*Diagnosis*	Multiple Randomized Controlled Trials, or Cross-sectional Studies with verification by reference (gold) standard, or Systematic Reviews of such studies

**II**	*Effectiveness*	One Randomized Controlled Trial
	
	*Diagnosis*	One Randomized Controlled Trial, or one Cross-sectional Study with verification by reference (gold) standard

**III**	*Effectiveness*	Multiple Controlled nonrandomized Studies or Systematic Reviews of such studies
	
	*Diagnosis*	Multiple Cross-sectional Studies with incomplete & unbalanced verification with reference (gold) standard

**IV**	*Effectiveness*	Other studies
		
	*Diagnosis*	

**V**	*Effectiveness*	SOSORT Consensus with more than 90% of agreement
		
	*Diagnosis*	

**VI**	*Effectiveness*	SOSORT Consensus with 70 to 89% of agreement
		
	*Diagnosis*	

Questions on Effectiveness (treatment results) and Diagnosis (assessment) have been considered

**Table 2:** Strength of Recommendations grading used in these Guidelines.

Strength of recommendation	Meaning
**A**	it must be applied widely and to all patients with this specific need

**B**	it is important, but can be applied not to all patients with this specific need

**C**	less important, it can be applied on a voluntary basis

**D**	very low importance

### Target users of the Guidelines

Users of these Guidelines are meant to be all professionals involved in the Conservative Treatment of Scoliosis, but they also should serve as reference for patients.

### Updates

Since these Guidelines have been produced in 2011, they will be fully updated by SOSORT between 2016 and 2021. If important changes in practice will intervene before, an update could be decided by the SOSORT Board to be published before that date.

### Applicability

These Guidelines will be published in the Internet Open Access Journal "Scoliosis" http://www.scoliosisjournal.com. This is the most important way to ensure their accessibility to the worldwide community of Scoliosis conservative professionals. Moreover, this will guarantee visibility to the patients. The Consensus process, involving professionals from all over the world, should provide an objective document that a wide variety of interested organizations and third party payers may review to gain insight into the treatment modalities. In the meantime, single national adaptations should eventually be considered. The document in itself should serve ad the basis for these national documents.

Translations in different languages have been already planned, including: French, German, Greek, Italian, Japanese, Polish, Spanish. These translations will be published in the Official SOSORT website: http://www.sosort.org. Moreover, process for National Organizations approvals have been planned, and will be reported in the next Edition of these Guidelines.

## General information on idiopathic scoliosis

### Definitions

Scoliosis is a general term comprising a heterogeneous group of conditions consisting in changes in the shape and position of the spine, thorax and trunk. The name, believed to have been introduced by Hippocrates (scolios, which means crooked or curved) [5] and used by Galen (scoliosis), means an abnormal lateral spinal curvature. Today, scoliosis is known not to be limited only to the frontal plane, and can be defined as a "**three-dimensional torsional deformity of the spine and trunk**" [6-8]: it causes a lateral curvature in the frontal plane, an axial rotation in the horizontal one, and a disturbance of the sagittal plane normal curvatures, kyphosis and lordosis, usually, but not always, reducing them in direction of a flat back.

"Structural scoliosis", or just scoliosis, must be differentiated from "functional scoliosis", that is a spinal curvature secondary to known extraspinal causes (e.g. shortening of a lower limb or paraspinal muscle tone asymmetry). It is usually partially reduced or completely subsides after the underlying cause is eliminated (e.g. in a recumbent position). Functional scoliosis is not the subject of this paper.

The term Idiopathic Scoliosis was introduced by Kleinberg (1922) (ref), and it is applied to all patients in which it is not possible to find a specific disease causing the deformity; in fact, it appears in apparently healthy children, and can progress in relation to multiple factors during any rapid period of growth. By definition, idiopathic scoliosis is of unknown origin and is probably due to several causes. Etiopathogenetically, the spinal deformity caused by idiopathic scoliosis may be defined as a **sign of a syndrome with a multifactorial etiology **[9-13]. Nearly always, scoliosis manifests as a solitary deformity, but further investigation may reveal other significant subclinical signs [14,15]. Idiopathic Scoliosis has been described as a torsional deformity of the spine, which combines a translation and rotation of a variable number of vertebrae, changing the 3D geometry of the spine [16-18]. Structural and sometimes a geometrical flat back is seen often, but the geometry of the spine in the lateral radiograph is highly variable. Trunk deformity and back asymmetry correlates with the spinal deformity, but there can be significant discrepancies in some cases [19].

The curvature in the frontal plane (AP radiograph in upright position) is limited by an 'upper end vertebra' and a 'lower end vertebra', taken both as a reference level to measure the Cobb angle. The Scoliosis Research Society (SRS) suggests that the diagnosis is confirmed when the Cobb angle is 10° or higher and axial rotation can be recognized. Maximum axial rotation is measured at the apical vertebra. However, structural scoliosis can be seen with a Cobb angle under 10° [20], with a potential for progression. Progression is more common in girls during the growth spurt at puberty and then it is called progressive Idiopathic Scoliosis. When untreated, it may lead to severe trunk deformities, which limit the capacity and functional biomechanics of the chest, exercise capacity, general fitness and ability to work, all factors related with impairment on quality of life.

### Epidemiology

In approximately 20% of cases, scoliosis is secondary to another pathological process. The remaining 80% are cases of idiopathic scoliosis. Adolescent idiopathic scoliosis (AIS) with a Cobb angle above 10° occurs in the general population in a wide range from 0.93 to 12% [21-38]: two to three percent is the value the most often found in the literature, and it has been suggested that epidemiology changes according to latitude [24,39].

Approximately 10% of these diagnosed cases require conservative treatment and approximately 0.1-0.3% require operative correction of the deformity. Progression of AIS is much more frequently seen in females. When the Cobb angle is 10 to 20°, the ratio of affected girls to boys is similar (1.3:1), increasing to 5.4:1 for Cobb angles between 20 and 30°, and 7:1 for angle values above 30° [40,41]. If the scoliosis angle at completion of growth exceeds a"critical threshold" (most authors assume it to be between 30° and 50°), there is a higher risk of health problems in adult life, decreased quality of life, cosmetic deformity and visible disability, pain and progressive functional limitations [41,42].

### Etiology

The etiopathogenesis of scoliosis has not been elucidated. The causes of scoliosis are being sought in congenital or acquired disorders of vertebral structure. Patients with this type of deformity are usually noted to suffer from such co-existent abnormalities as asymmetrical structure of the brain stem, sensory and balance impairment, disorders of blood platelet and collagen function [3-5]. The role of genetic factors in the development of spinal axial disorders is also emphasised and is confirmed by the tendency of scoliosis to run in families, with researchers suggesting a hereditary disorder of oestrogen receptor structure and function [6].

Numerous authors indicate that the causes of scoliosis are systemic disorders of, among others, mucopolysaccharide and lipoprotein synthesis. In the 1990s a group of researchers under the guidance of Dubousset [7-9] proposed that scoliosis develops as a result of melatonin synthesis disorder. They produced spinal curvatures in chickens via pinealectomy and later ameliorated the melatonin deficiency to find decreased incidence of scoliosis in the animals. Machida reported reduced serum melatonin levels in girls with rapidly progressive idiopathic scoliosis [8]. His finding has been questioned by other authors, who found no differences between melatonin levels in scoliotic girls and those in a healthy control group. Currently, melatonin is attributed only a limited role in scoliosis pathogenesis [10]. The possible role of melatonin in scoliosis etiology is also discussed in connection to age at menarche in different geographic latitudes. [24]

According to more recent studies, calmodulin may disturb melatonin levels. Kindsfater [43] assessed calmodulin levels in order to determine the risk of curve progression. Basing on this hypothesis, melatonin plays a secondary role in the spontaneous induction of scoliosis. It is a consequence of interaction with calmodulin, a protein that has receptors for calcium ions and is thus able to influence the contractility of skeletal muscles; it can also be found in blood platelets (its level in platelets was higher in patients with scoliotic progression rates of more than 10° over 12 months) [11]. Other authors have evaluated the possibility that gene variants of IL-6 and MMPs might be associated with scoliosis and suggests that MMP-3 and IL-6 promoter polymorphisms constitute important factors for the genetic predisposition to scoliosis. Association Between IL-6 [44].

All in all, the etiology of scoliosis has not been fully elucidated [12,13]. Based on the variety of opinions on idiopathic scoliosis development, we can assume a multifactorial origin. The opinions presented above are supplementary rather than mutually exclusive. At the same time they explain the complex determinants of and relationships between disorders of spinal development in children and adolescents.

### Natural history

Idiopathic scoliosis (IS) may develop at any time during childhood and adolescence. It is most common in periods of growth spurt-between the ages of 6 and 24 months, 5 and 8 years and 11 and 14 years of life [2]. The rate of development of spinal curvature changes the most rapidly at the beginning of puberty [23,24]. According to the Tanner scale, which assesses tertiary sex characteristics, this period corresponds to stage S2 and P2 in girls, and T2 and P2 in boys [25]. The pubertal growth spurt begins with accelerated longitudinal growth of limbs, which causes a temporary disproportion of the body (long limbs and short trunk). Then longitudinal growth is seen in the axial skeleton. It is the period of the most marked progression of IS. After approx. 2/3 of the period of pubescent growth spurt, girls experience menarche, which indicates a slow, gradual decrease in the risk of scoliosis progression:

There is a much lower potential for progression of idiopathic scoliosis after the spinal growth is complete. In adulthood, IS may intensify as a result of progressive osseous deformities and collapsing of the spine. This phenomenon is reported especially in scoliosis that is more severe than 50°, while the risk of progression starts to increase as the curve grows above 30° [26,30,31,42]; less severe idiopathic scoliosis curves often remain stable. Nevertheless, the natural history of adult scoliosis is not well known to date, and it is still possible the progression can have some peak periods [45]. A "de novo" scoliosis has been recognized as a possible form in adulthood [46].

### Classifications

During the years, many different classifications of idiopathic scoliosis have been proposed, but not all of them are either relevant for conservative care, or currently used beyond research purposes. In Table 3 we present the most relevant clinical conservative practices used in clinical practice, with a short discussion that follows.

**Table 3:** Classifications of idiopathic scoliosis.

Chronological		Angular			Topographic		
**Age at diagnosis (years.months)**		**Cobb degrees**				**Apex**	
						
						**from**	**to**

Infantile	0-2.11	Low	Low	5-15	Cervical	-	Disc C6-7
		
Juvenile	3-9.11		Low to moderate	16-24	Cervico-thoracic	C7	T1

Adolescent	10-17.11	Moderate	Moderate	25-34	Thoracic	Disc T1-2	Disc T11-12
		
Adult	18-		Moderate to severe	35-44	Thoraco-lumbar	T12	L1

		Severe		45-59	Lumbar	Disc L1-2	-
		
		Very severe		60 or more			

#### Chronological

It has been proposed by James [2], that scoliosis should be classified based on the age of the child at which the deformity was diagnosed (Table 3). This classification is important since the longer the period between diagnosis of scoliosis and completion of growth by the developing child, the greater the risk of developing a more severe and complicated deformity.

Today the general term "Early onset scoliosis" is sometimes used to classify together Infantile and Juvenile scoliosis, but we prefer the James classification, due to the fact that infantile scoliosis has a different prognosis. In fact there are congenital postural scoliosis curves diagnosed in newborns, as a component of a syndrome usually resulting from intrauterine compression caused by malposition of the fetus during pregnancy, and it is an exception to the rule. Such curvatures are not three-plane deformities and usually undergo spontaneous remission. As the range of hip motion is often asymmetrical and the child prefers to rest their head on one side only, exercises and correction of body position are usually employed. Examination usually reveals gradual remission of the curvature in these infants, and such scoliosis curves may thus be categorised as regressive [17].

#### Angular

The angle of scoliosis measured on the standing frontal radiograph according to the Cobb method is one of the decisive factors in managing idiopathic scoliosis, and it is directly correlated to all therapeutic decisions. Many different classifications have been proposed based on these angular measurements, but no one system today has widespread validity. Nevertheless, there is an agreement on some thresholds [41,42,47-49]:

• under 10° of scoliosis, the diagnosis of scoliosis should not be made;

• over 30° of scoliosis the risk of progression in adulthood increases, as well as the risk of health problems and reduction of quality of life;

• over 50° there is a consensus that it is almost certain that scoliosis is going to progress in adulthood and cause health problems and reduction of quality of life.

From these thresholds, and taking into account that the recognised measurement error in measuring Cobb angles is 5° [50-55], very important decisions are made. These include the generally recognised threshold for surgery (45-50°), and the aims of conservative treatment that we will describe below. We propose here a classification useful for conservative physicians and as a way to discuss therapeutic options with the patients (Table 3): it comes from the idea that there is a continuum from one stage to the other, and that the 5° measurement error must be taken into account.

#### Topographic

The remaining most common classifications of idiopathic scoliosis are based on the anatomical site of the spinal deformity in the frontal plane only. A classification developed by Ponseti [56] (based on Schulthess work [57]) distinguishes four major types of scoliosis: thoracic, lumbar, thoraco-lumbar and S-shaped. This classification is the most traditional and used both in conservative treatment and in the pre-operative classification of scoliosis [58], and is reported in Table 3. Two other classification systems of idiopathic scoliosis based on the anatomical site of spinal deformity have been proposed and used in preoperative planning [59-63]. Since these Guidelines deal with conservative treatment, they are not considered here. In the clinical setting of rehabilitation and bracing other classifications have been proposed, but they have not yet become standards [64-68]; moreover, some 3D classifications have been published as well [69-75], but they are far from being validated for clinical everyday application.

## Evidence-Based Clinical Practice approach to Idiopathic Scoliosis

### Goals of conservative treatment

#### General Goals

SOSORT has published in the *Scoliosis Journal *a Consensus Paper titled "Why do we treat adolescent idiopathic scoliosis? What do we want to obtain and to avoid for our patients. SOSORT 2005 Consensus paper" [42] that can serve as reference for specific insights on this topic. In this paper, the most general goals of treatment can be found (Table 4).

**Table 4:** Goals of treatment according to the SOSORT Consensus paper [42].

Rank	Aim	Percentage of responders
1	Esthetics	100%
2	Quality of life	91%
3	Disability	91%
4	Back Pain	87%
5	Psychological well-being	84%
6	Progression in adulthood	84%
7	Breathing function	84%
8	Scoliosis Cobb degrees	84%
9	Need of further treatments in adulthood	81%

Only the goals that reached 80% of agreement are listed here, starting from the most important. The column "Percentage of responders" refers to those that considered each outcome relevant during the Consensus Conference.

The goals of conservative treatment of idiopathic scoliosis may be divided into two groups: morphological and functional. The first aspect influences aesthetics (that has been proposed as the first goal of treatment by SOSORT experts), while both aspects determine patients' quality of life, psychological well-being, and disability (the second to fourth goals according to SOSORT experts) [42]. The basic objectives of comprehensive conservative treatment of Idiopathic Scoliosis are:

1. to stop curve progression at puberty (or possibly even reduce it),

2. to prevent or treat respiratory dysfunction,

3. to prevent or treat spinal pain syndromes,

4. to improve aesthetics via postural correction,

##### To stop curve progression at puberty (orpossibly even reduce it)

It is believed that it is impossible to fully eradicate idiopathic scoliosis with conservative treatment techniques available at present. It is possible and usually sufficient to prevent further progression, even if recent research papers conducted according to the SRS criteria have shown that it is also possible to obtain some amount of curve correction [76-79].

##### To prevent or treat respiratory dysfunctions

The morphological aspect of the deformity is closely related to the functional aspect. Depending on its degree and location, the curvature affects respiratory function. The most prominent changes within the respiratory system are produced by curvatures of the thoracic spine.

##### To prevent or treat spinal pain syndromes

Scoliotic adults suffer from spinal pain, which they experience more frequently than non-scoliotic adults. Statistically significant differences are already noted in people between 20 and 30 years of age. In a follow-up study of over 40 years duration, three-fold higher prevalence of chronic pain-related complaints and over twenty-fold higher incidence of severe and darting pain in a group of people with untreated idiopathic scoliosis compared to a control group. The occurrence of pain-related complaints is probably multifactorial in origin [80-87].

##### To improve the appearance via postural correction

Quality of life is significantly affected by aesthetic sensation and acceptance of one's appearance. Therefore, visual correction of a scoliosis related external trunk deformity is an important issue in conservative treatment. The assessment of therapeutic outcomes may be based on subjective visual assessment, on specially developed indices of visual evaluation or on parameters of surface topography assessment [19,88,89].

#### Specific goals of conservative treatment during growth

It is possible to define specific goals of conservative treatment of single patients during growth: these can be set according to the starting point (x-ray before treatment). These goals should be considered as a dynamic tool, to be adapted during treatment according to the change in the deformity, compliance of the patient, therapies proposed and so on. In this respect, we can define the following possibilities:

• Absolute goal: these are the bottom line of conservative treatment. If not anything else, at least these goals should be reached.

• Primary goal: these are the "best possible" goals for patients starting treatment in each specific clinical situation

• Secondary goals: these are the compromise goals that come when it becomes clear that it is not possible to reach the primary goals

According to this approach, SOSORT has reached a Consensus (Strength of Evidence VI-Strength of Recommendation C) shown in Table 5. This table has been organized with a minimum and a maximum of primary and secondary goals that can be reached for each clinical situation. The absolute goals are similar for all patients in every clinical situation: avoid fusion surgery. A first approach to this problem, developing a similar scheme, has been proposed in 2007 [90]: these goals were applied in some studies [77,90] and proved to be useful. Accordingly, we propose here these goals of treatment to be applied in clinical studies of conservative treatment of idiopathic scoliosis.

**Table 5:** Specific aims of conservative treatment during growth (Strength of Evidence VI-Strength of Recommendation C)

		Adolescent Idiopathic Scoliosis up to 45°	Adolescent Idiopathic Scoliosis over 45°	Infantile and JuvenileIdiopathic Scoliosis
***Radiographic aims***	**Primary**	Below 25°	Below 35°	Below 25°
	
	**Secondary**	Below 35°	No progression	Below 50°

***Main aims***		Avoid surgery Improve aesthetics and quality of life Reduce disability and pain		

Notes and definitions

• Final results depend on the characteristics of the disease (progressive potential) and not only on the quality and quantity of treatment (that rely on the action of the whole team: physician, orthotist, therapist, family and patient)

• **Goals of treatment**: what treating team would like to achieve in front of a specific clinical situation.

• **Main aims**: pursued in all cases beyond Cobb degrees results

• **Primary aims**: pursued at start of treatment, but not possible in all cases

• **Secondary aims**: to be pursued if primary aims are not achievable, but also secondary aims are not always possible

### Evidence-Based Clinical Practice approach

This section is constituted mainly by a Practical Approach Scheme (PAS) (Table 6) that has been prepared through the Consensus Procedure reported in Appendix (Additional File 1). The PAS constitutes a real Evidence Based Clinical Practice Approach to Idiopathic Scoliosis. The Strength of Evidence of PAS is VI, while the Strength of Recommendation is B.

**Table 6:** Practical Approach Scheme (PAS) for an Evidence Based Clinical Practice approach to Idiopathic Scoliosis (Strength of Evidence VI-Strength of Recommendation B).

		Cobb degrees	0-10 + hump	11-15	16-20	21-25	26-30	31-35	36-40	41-45	46-50	Over 50
**Infantile**		*Min*	Ob6	Ob6	Ob3	SSB	SSB	SSB	SSB	SSB	PTRB	FTRB
		
		*Max*	Ob3	Ob3	PTRB	FTRB	FTRB	FTRB	FTRB	FTRB	Su	Su

**Juvenile**		*Min*	Ob3	Ob3	Ob3	SSB	SSB	SSB	PTRB	PTRB	PTRB	FTRB
		
		*Max*	PSE	PSE	PTRB	FTRB	FTRB	FTRB	FTRB	FTRB	Su	Su

**Adolescent**	*Risser 0*	*Min*	Ob6	Ob6	Ob3	PSE	PSE	SSB	PTRB	PTRB	PTRB	FTRB
		
		*Max*	Ob3	PSE	PTRB	FTRB	FTRB	FTRB	FTRB	FTRB	Su	Su
	
	*Risser 1*	*Min*	Ob6	Ob6	Ob3	PSE	PSE	SSB	PTRB	PTRB	PTRB	FTRB
		
		*Max*	Ob3	PSE	PTRB	FTRB	FTRB	FTRB	FTRB	FTRB	Su	Su
	
	*Risser 2*	*Min*	Ob8	Ob6	Ob3	PSE	PSE	SSB	SSB	SSB	SSB	FTRB
		
		*Max*	Ob6	PSE	PTRB	FTRB	FTRB	FTRB	FTRB	FTRB	Su	Su
	
	*Risser 3*	*Min*	Ob12	Ob6	Ob6	Ob6	PSE	SSB	SSB	SSB	SSB	FTRB
		
		*Max*	Ob6	PSE	PTRB	FTRB	FTRB	FTRB	FTRB	FTRB	Su	Su
	
	*Risser 4*	*Min*	No	Ob6	Ob6	Ob6	Ob6	Ob6	Ob6	Ob6	SSB	FTRB
		
		*Max*	Ob12	PSE	PTRB	FTRB	FTRB	FTRB	FTRB	FTRB	Su	Su
	
	*Risser 4-5*	*Min*	No	Ob6	Ob6	Ob6	Ob6	Ob6	Ob6	Ob6	SSB	FTRB
		
		*Max*	Ob12	PSE	PTRB	FTRB	FTRB	FTRB	FTRB	FTRB	Su	Su

**Adult**	*No pain*	*Min*	No	No	No	No	No	No	No	No	Ob12	Ob12
		
		*Max*	Ob12	Ob12	Ob12	Ob12	Ob12	Ob12	Ob12	Ob12	Ob6	Ob6
	
	*Chronic Pain*	*Min*	No	PSE	PSE	PSE	PSE	PSE	PSE	PSE	PSE	PSE
		
		*Max*	PTRB	PTRB	PTRB	PTRB	PTRB	Su	Su	Su	Su	Su

**Elderly**	*No pain*	*Min*	No	No	No	No	No	No	No	No	Ob12	Ob12
		
		*Max*	Ob12	Ob12	Ob12	Ob12	Ob12	Ob12	Ob12	Ob12	Ob6	Ob6
	
	*Chronic Pain*	*Min*	No	PSE	PSE	PSE	PSE	PSE	PSE	PSE	PSE	PSE
		
		*Max*	PTRB	PTRB	PTRB	PTRB	PTRB	PTRB	PTRB	PTRB	Su	Su
	
	*Decompensation*	*Min*	No	No	PSE	PSE	PSE	PSE	PSE	PSE	PSE	PSE
		
		*Max*	PTRB	PTRB	PTRB	PTRB	PTRB	PTRB	PTRB	PTRB	Su	Su

For each single clinical situation reported in any single cell, a minimum and a maximum strength of treatment is listed. The graduation of strength of treatments have been reported in the Strength of Treatments Scheme in Table 8. Consequently, all treatments included between the minimum and maximum can be considered for that specific clinical situation.

Obs 36/12/8/6/4: Observation every 36/12/8/6/4 months; PSE: Physiotherapeutic Specific Exercises; NTRB: Night-time Rigid Bracing (8-12 hours); SIR: Inpatient rehabilitation; SB: Soft bracing; PTRB: Part-Time Rigid Bracing (12-20 hours); FTRB: Full-time Rigid bracing (20-24 hours) or cast; Su: Surgery.

This paper also presents a Strength of Treatments Scheme (STS) (Table 7) that reports all the possible treatments that can be proposed for Idiopathic Scoliosis graduated from the least to the most demanding (both in terms of burden on the patient, and possible efficacy). In addition, the STS is Consensus based (Strength of Evidence V-Strength of Recommendation B). Starting from the STS it is possible to state, for each single clinical situation of the PAS, a minimum and a maximum of possible treatments that could be proposed: consequently all treatments that in the STS are reported between this minimum and maximum can be considered for that specific clinical situation.

**Table 7:** Strength of Treatments Scheme (STS) (Strength of Evidence V-Strength of Recommendation B): it reports all the possible treatments that can be proposed for Idiopathic Scoliosis graduated from the less to the most demanding (both in terms of burden on the patient, and possible efficacy).

Min	Treatment	Abb	Notes
0	Nothing	No	

1	Observation every 36 months	Ob36	- Observation is clinical evaluation and not x-ray everytime
	
2	Observation every 12 months	Ob12	- X-rays are usually performed once every two clinical evaluations, unless otherwise justified in the opinion of a clinician specialized in conservative treatment of spinal deformities
	
3	Observation every 8 months	Ob8	
	
4	Observation every 6 months	Ob6	
	
5	Observation every 3 months	Ob3	

6	Physiotherapeutic Specific Exercises (outpatient)	PSE	- The term "Physiotherapeutic" added to "Physiotherapeutic Specific Exercises" does not designate an exclusive professional proposing the exercises, but the general approach to the patient, that goes beyond the simple execution of exercises
	
7	Night-time Rigid Bracing (8-12 hours)	NTRB	- According to the actual evidence it is not possible to define which treatment is more effective than the others between PSE (#6) and PTRB (#10), consequently the progressive numbers should be regarded only as a tool to be applied to the Practical Approach table and not as a classification approved by SOSORT members
	
8	Inpatient rehabilitation	SIR	
	
9	Specific Soft Bracing	SSB	

10	Part-Time Rigid Bracing (12-20 hours)	PTRB	The use of a rigid brace always imply the associated use of Physiotherapeutic Specific Exercises
	
11	Full-time Rigid bracing (20-24 hours) or cast	FTRB	

12	Surgery	Su	

Max			

Min: minimum; Max: maximum; Abb: abbreviation

**Figure 1:**
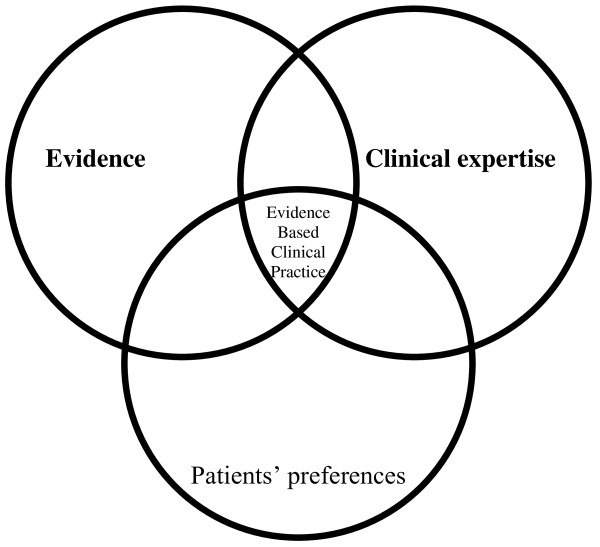
**Graphical representation of Evidence Based Clinical Practice as the meeting point among evidence (coming from Evidence Based Medicine), individual physician's clinical expertise and patients' preferences**.

The PAS has some main characteristics that constitute its strength and justification:

• It constitutes the way we have chosen to resolve the differences among the various clinicians in their everyday clinical approach, to be able to state what is presumably totally wrong (above the maximum: overtreatment-below the minimum: undertreatment) according to the actual conservative treatment knowledge.

• It reports a real everyday approach, since all clinicians usually chose from quite a wide panel of choices when treating a single patient; the final decision comes after discussion with the patient, and weighting of the various risk factors involved in the clinical situation. In fact, the PAS has been developed looking at the "Step by Step" Sibilla's theory [78,91-94]: for each single patient it is mandatory to chose the correct step of treatment, where the most efficacious is also the most demanding. Accordingly, coming to a wrong decision means facing one of the two main mistakes in conservative treatment of idiopathic scoliosis, overtreatment (too much burden on the patient) or undertreatment (not enough efficacy).

• Evidence-Based Clinical Practice is by definition the best integration between the knowledge offered by Evidence-Based Medicine, individual clinical expertise and patients' preferences (Figure 1) [95-98]. Consequently, a single patients treatment by different clinicians, even when faced with the identical clinical situation, can vary either because of the patient preferences or because of the specific expertise of the clinician. This has the final consequence that it will never be possible to state definitively what is the only right approach to a clinical situation, but always a range of situations need to be considered.

In the PAS it has been accepted that single conservative expert physicians treating idiopathic scoliosis patients can move up and down in the same range of treatments, but also to the right or to the left (i.e. changing to a more or less demanding clinical situation, here identified as a column of the PAS), according to the presence or absence of specific risk factors that have been listed at the bottom of PAS.

Below we briefly list and describe the different treatments considered in the PAS and listed in the STS. A short description of the various risk factors of progression is provided as well.

### Conservative treatments

All these treatment approaches are listed in the STS (Table 7) and will be presented from the less to the most demanding and possibly efficacious. For more insight it is possible to look at the Brace Technology and Rehabilitation Schools for Scoliosis Series [99,100] published by the journal *Scoliosis*. Moreover, more specific detail can be found in the Consensus paper on Terminology recently produced by SOSORT [101].

**Nothing (No)**: No treatment is needed.

**Observation (Ob)**. It is the first step of an active approach to idiopathic scoliosis and it is constituted by regular clinical evaluation with a specific follow-up period. Timing of this follow-up can range from 2-3 to 36-60 months according to the specific clinical situation. Clinical evaluation does not mean performing x-rays everytime: x-rays are usually performed during alternate clinical evaluations.

**Physiotherapeutic Specific Exercises (PSE)**. They include all forms of outpatient physiotherapies that have proven efficacy, that will gradually be published in the Rehabilitation Schools for Scoliosis Series [100] in the journal *Scoliosis*. They have been listed in the 3^rd ^part of these Guidelines. The frequency of therapeutic sessions depends on the techniques, cooperation and the ability of the patient to carry out the treatment with the assistance of caregivers. At times, it can be conducted daily or several times a week. Long-term outpatient physiotherapy sessions most often take place 2-4 times a week if the patient is willing to co-operate fully. The actual form of exercise depends mainly on the character of the selected therapeutic method.

**Special Inpatient Rehabilitation (SIR)**. This is a special exercise method used on an in-patient basis (hospital department, sanatorium or a similar form of health care). SIR is advised by some schools especially at the beginning of exercise treatment in order to teach the patient and his caregivers how to perform exercises properly.

**Bracing**: using a brace (a corrective orthosis) for a specified period of time each day to correct scoliosis in three planes (3D). It is used for a period necessary to obtain and maintain the therapeutic outcome. The therapeutic outcome is mainly the halting of scoliosis progression. In some cases it is possible to correct the scoliosis while in others the progression rate can only be slowed down before elective surgery. According to SOSORT, the use of a rigid brace always implys the additional use of exercises when out of the brace. Bracing includes:

• **Night Time Rigid Bracing **(8-12 hours per day) **(NTRB)**: wearing a brace mainly in bed.

• **Soft Bracing (SB)**: it includes mainly the SpineCor brace [102,103], but also other similar designs [104,105]

• **Part Time Rigid Bracing **(12-20 hours per day) **(PTRB)**: wearing a brace mainly outside school and in bed.

• **Full Time Rigid Bracing **(20-24 hours per day) **or cast (FTRB)**: wearing a brace all the time (at school, at home, in bed, etc.). Casts have been included here as well. Casts are used by some schools as the first stage to achieve correction to be maintained afterwards with rigid brace [106-108]; others propose casting only in worst cases [92,93,109,110]; a cast is considered a standard approach in infantile scoliosis [111]. Recently, a new brace has been developed that has been claimed to achieve same results as casting [77,112,113].

A common feature of all forms of conservative treatment is the need to actively involve the patient and caregivers [114]. Therefore education, psychotherapy, systematic monitoring of outcomes, assessment of patient's co-operation, and verification and modification of methods in the course of the therapy are crucial elements of conservative treatment. In order to achieve the best possible outcome, conservative treatment should be conducted by an experienced therapeutic team including a physician, a physiotherapist, an orthotist and possibly a psychologist [114]. Support groups and internet forums are also important in conservative treatment.

### Prognostic factors

Using the PAS it is mandatory to include prognostic factors so to move properly between the minimum and maximum strength of treatment. The following factors have been suggested as possible determinants of a higher risk of scoliosis progression: positive family history, laxity of skin and joints (connective tissue defect), flattening of physiological thoracic kyphosis (impedes efficient bracing), angle of trunk rotation exceeding 10°, growth spurt.

Bunnell reported that the risk of progression at the beginning of puberty is 20% in 10° scoliosis, 60% in 20° scoliosis, and as much as 90% in 30° scoliosis [47,115]. At the age of peak height growth (13 years of osseous age in girls) the risk of progression is 10%, 30% and 60%, respectively. During the final stage of puberty (at least Risser grade II) the risk of deformity progression becomes considerably lower, falling to 2% in 10° scoliosis, 20% in 20° scoliosis and 30% in 30° scoliosis. The prognosis regarding IS progression seems to be more optimistic for boys. [116].

The risk of progression rises with more severe loss of physiological thoracic kyphosis and higher Cobb angles at diagnosis of IS, even if the lateral spine profile of mild (10°-20°) scoliotic curves was found to be similar to the lateral spine profile of their healthy controls [117]. Evidence that thoracic hypokyphosis, by facilitating axial rotation, could be viewed as being permissive (a compensatory mechanism), rather than as etiological factor, in IS pathogenesis has also been provided [118].

The pathologic mechanism of progression in an IS curve is nicely described in some recently published papers [12,119-121]. The factors which progression is attributed to are: the effect of gravity, the muscle action, the reactive forces causing increased lordosis, the human gait, and the growth induced torsion. The intervertebral disc could be included as an additional morphological factor involved in the progression of an IS curve [7,100,122].

The determination of the risk of idiopathic scoliosis progression has recently been made possible through genetic assessment, with 53 loci identified [48,123]. The determination of the polymorphism of selected genes is supposed to facilitate the assignment of a patient to a progressive or stable group [124-126]. A prognostic genetic test has been developed as well [126]. Although these initial results have been promising, great caution is still advised at this stage of the research, while we wait for more stronger proof of efficacy.

Finally, during recent years there have been several prognostic formulas that have been proposed [127-129]. The previous SOSORT guidelines [1] were based on the Lonstein and Carlson factor of progression [129] for the assessment of the risk of idiopathic scoliosis. Since there are no formulas that have been applied in specific studies after their development to verify their real efficacy, we do not apply them in these Guidelines.

Beyond all this discussion, the actual SOSORT Consensus suggests that we consider the following prognostic factors: family history, proven progression, decompensation, short curve, pain, Scoliscore, flat back, and esthetic impact.

## Brace treatment

### Methods

In February 2011 we performed a search of Medline from its inception, with no language limitations. We used the following search strategies:

• *"Braces"[Mesh] AND "Scoliosis"[Mesh] AND (hasabstract[text] AND (Clinical Trial[ptyp] OR Meta-Analysis[ptyp] OR Practice Guideline[ptyp] OR Randomized Controlled Trial[ptyp] OR Review[ptyp])) *(155 papers).

• *("Scoliosis/therapy"[Mesh]) AND "Braces"[Mesh] AND compliance *(78 papers)

• *"Scoliosis"[Mesh] AND "Braces"[Mesh] AND ("infant, newborn"[MeSH Terms] OR "infant"[MeSH Terms:noexp] OR "child, preschool"[MeSH Terms]) *(183 papers)

We selected from the titles a total of 224 papers and, looking at the abstracts, 102 were selected and retrieved in full text. We also searched: the abstracts of all SOSORT Meetings, from the first one in 2003 to 2010; the personal files and knowledge of all the authors; the papers retrieved with all the other searches listed in these Guidelines; the references sections of all retrieved papers. The selection criteria used in all these searches were: pertinence for the topic "Brace treatment"; presence of the abstract; numerical results in relation to scoliosis; retrievability in full text; all languages.

### Results

SOSORT has published in *Scoliosis Journal *two Consensus Papers on bracing titled "SOSORT consensus paper on brace action: TLSO biomechanics of correction (investigating the rationale for force vector selection)" [130], and "Guidelines on "Standards of management of idiopathic scoliosis with corrective braces in everyday clinics and in clinical research": SOSORT Consensus 2008" [114]: they can serve as reference for specific insights.

#### Efficacy in adolescents

Recently a Cochrane review [131,132] has been published, that found that there is very low quality evidence in favor of using braces, making generalization very difficult. This review included:

• one multicenter prospective international observational study that provided very low quality evidence in favor of the efficacy of bracing [133]: Nachemson evaluated 240 patients with thoracic or thoracolumbar curves between 25° and 35°, aged between 10 and 15 years, of which 129 were only observed and 111 treated with thoracolumbar braces. Progression of 6 or more degrees at 2 radiographic follow-ups to the first visit was considered an index of failure of the selected treatment (observation versus brace treatment). At 4 years of follow-up, the success rate for brace treatment was 74% (range, 52--84%), whereas the rate for observation was 34% (range, 16--49%).

• a randomized controlled trial that demonstrated with very low quality evidence that a plastic TLSO brace is more effective than an elastic brace [134]. Wong randomized forty-three subjects to SpineCor or rigid orthosis group. Although it has been stated that the authors where not trained to fit the SpineCor brace [135] the authors concluded that 68% of the subjects in the SpineCor group and 95% of the subjects in the rigid orthosis group did not show curve progression, with a significant difference. The 2 groups had similar responses to a patient acceptance questionnaire.

The Cochrane review concluded that further research could change the actual results and our confidence in them; in the meantime, patients' choices should be informed by multidisciplinary discussion. Future research should focus on short- and long-term patient-centered outcomes, in addition to measures such as Cobb angles. RCTs and prospective cohort studies should follow both the Scoliosis Research Society and Society on Scoliosis Orthopedic and Rehabilitation Treatment criteria for bracing studies.

In fact, beyond the previously reported papers, the SRS defined some methodological criteria to be followed during brace cohort studies [136]. The optimal inclusion criteria consist of: age 10 years or older when brace is prescribed, Risser 0-2, primary curve angles 25 degrees-40 degrees, no prior treatment, and, if female, either premenarchal or less than 1 year postmenarchal. Assessment of brace effectiveness should include: (1) the percentage of patients who have < or = 5 degrees curve progression and the percentage of patients who have > or = 6 degrees progression at maturity, (2) the percentage of patients with curves exceeding 45 degrees at maturity and the percentage who have had surgery recommended/undertaken, and (3) 2-year follow-up beyond maturity to determine the percentage of patients who subsequently undergo surgery. All patients, regardless of subjective reports on compliance, should be included in the results (intent to treat). Every study should provide results stratified by curve type and size grouping. Cohort studies respecting the SRS criteria can be considered of high methodological quality. Until now 6 papers have been published with these characteristics [76,78,137-139].

Together with these criteria, SOSORT offered the "Standards of management of idiopathic scoliosis with corrective braces in everyday clinics and in clinical research" [114], that include 14 recommendations, grouped in 6 Domains (Experience/competence, Behaviours, Prescription, Construction, Brace Check, Follow-up). Cohort studies using the SOSORT criteria can be considered of high quality in terms of patient and treatment management. Until now 2 papers have been published with these characteristics [76,78].

Looking at the papers published using the SRS and/or SOSORT criteria we found:

• Janicki et al [138], following the SRS criteria, retrospectively compared in an "intent-to-treat" analysis the effectiveness of the custom thoracolumbosacral (TLSO) worn 22 hours/day and the Providence orthosis worn 8-10 hours/night. There were 48 patients in the TLSO group and 35 in the Providence group. In the TLSO group, only 7 patients (15%) did not progress (< or = 5 degrees), whereas 41 patients (85%) progressed by 6 degrees or more, including the 30 patients whose curves exceeded 45 degrees. Thirty-eight patients (79%) required surgery. In the Providence group, 11 patients (31%) did not progress, whereas 24 patients (69%) progressed by 6 degrees or more, including 15 patients whose curves exceeded 45 degrees. Twenty-one patients (60%) required surgery.

• Coillard et al [137], following the SRS criteria, studied prospectively a cohort of 254 patients treated with the Dynamic SpineCor brace. Successful treatment (correction > 5° or stabilization ± 5°) was achieved in 165 patients of the 254 patients (64.9%). 46 immature patients (18.1%) required surgical fusion whilst receiving treatment. Two patients out of 254 (0.7%) had curves exceeding 45° at maturity.

• Negrini et al [78], following both the SRS and SOSORT criteria, retrospectively studied a cohort of 42 females and four males treated according to individual needs, with Risser casts, Lyon or SPoRT braces (14 for 23 hours per day, 23 for 21 h/d, and seven for 18 h/d at start). No patient progressed beyond 45 degrees, nor was any patient fused, and this remained true at the two-year follow-up for the 85% that reached it. Only two patients (4%) worsened, both with single thoracic curve, 25-30 degrees Cobb and Risser 0 at the start.

• Aulisa et al [76], following both the SRS and SOSORT criteria, retrospectively reviewed a cohort of fifty adolescent females with thoraco-lumbar curves treated with the Progressive Action Short Brace (PASB). Curve correction was accomplished in 94% of patients, whereas a curve stabilisation was obtained in 6% of patients. No patient required surgery, nor anyone progressed beyond 45°.

• Gammon et al [139], following the SRS criteria, compared treatment outcomes of 2 cohorts of patients treated via either a conventional rigid thoracolumbosacral orthoses (TLSO: 35 patients) or a SpineCor nonrigid orthosis (32 patients). No significant difference was found using the more strict outcome measure (< or = 5-degree curve progression) as the success rates were 60% for TLSO and 53% for SpineCor. Looking at patients who reached 45 degrees, the success rates were 80% for TLSO and 72% for SpineCor with no significant difference.

• Finally, Zaborowska-Sapeta et al [140], including the patients according to the SRS criteria, prospectively followed 79 patients treated with Cheneau brace. At one year after weaning the brace they found improvement in 25.3%, stabilization in 22.8%, progression of the Cobb angle up to below 50° in 39.2% and progression beyond 50° in 12.7%, the latter was considered surgical indication.

In summation, these papers show that: high variability among results of bracing is confirmed [76,78,137-140], and this is incredibly high mainly with rigid bracing [76,78,138-140]; even if soft braces [137,139] can have results better than [138], or at least comparable to [139], some types of rigid braces, the best results have been achieved with the last, when using SOSORT criteria [76,78,140]. It must also be noted that high variability can be found between different publications in the type of scoliosis treated, and thus a different outcome in treatment. A geographical distribution of different types of scoliosis should be taken in consideration and all results should be presented accordingly.

When it comes to previously published results, Dolan [141] performed a systematic review of the English literature: only studies written in English were included, if observation or a TLSO was evaluated and if the sample closely matched the current indications for bracing (skeletal immaturity, age 15 years or less, Cobb angle between 20° and 45°). Eighteen studies were included (3 observation only, 15 bracing). Despite some uniformity in surgical indications, the surgical rates were extremely variable, ranging from 1% to 43% after bracing, and from 13% to 28% after observation. When pooled, the bracing surgical rate was 23% compared with 22% in the observation group. It was concluded that, based on the evidence presented, one cannot recommend one approach over the other to prevent the need for surgery in AIS: the use of bracing relative to observation is supported by "troublingly inconsistent or inconclusive studies of any level".

Unfortunately, the inclusion criteria used by Dolan resulted in the exclusion of some retrospective papers already published at that time, since they had used exercises together with bracing [142-144]:

• Weiss [144] considered three hundred and forty-three scoliosis patients (females only) of various etiology, with a curvature of 33.4 degrees. Forty-one patients (11.95%) had had surgery. In patients with adolescent idiopathic scoliosis, the incidence of surgery was 7.3%.

• Rigo [142] considered 106 patients with curves on average of 30° at start, out of which 97 were followed up, and six cases (5.6%) ultimately underwent spinal fusion. A worst case analysis, which assumes that all nine cases that were lost to follow-up had operations, brings the uppermost number of cases that could have undergone spinal fusion to 15 (14.1%).

• Maruyama [143] reviewed 328 females with an average 32.4 degree Cobb angle. Surgery was recommended when curvature progressed to > 50 degrees. Twenty (6.1%) were treated with spinal fusion. The remaining showed no significant increase in magnitude of curvature.

In 2008 also Negrini [91] reported on surgery rates in curves over 30° at first evaluation, treated with brace and exercises: they were a subgroup of 28 out of 112 patientsof 23.4 Cobb degrees at the start of treatment. The rate of surgery was 1.9% (efficacy analysis), and 9.1% (worst case) versus 0.9% and 4.5% respectively in the whole group observed. All these studies, if included in the Dolan meta-analysis, would have changed the overall results in favor of bracing.

Some years ago, Rowe [145] conducted a meta-analysis to compare the consistency of outcomes among several of the oldest studies. Of a total of 1910 patients, 1459 received brace treatment, 322 electrostimulation, and 129 only observation. The weighted mean success rate was 0.39 for electrostimulation, 0.49 for observation, 0.60 for braces worn 8 hr daily, 0.62 for braces worn 16 hr daily, and 0.93 for braces worn 23 hr daily, the last of which was the statistically most efficacious treatment method. The most efficacious brace system was the Milwaukee brace vs. others, while the Charleston brace, which was worn only nighttimes, was the least successful, but yet statistically still better than observation alone.

##### Are there braces that are better than others?

In the literature there are very few studies comparing different braces. SOSORT experts, when facing the issue of trying to find a Consensus on the way to achieve the best possible correction through bracing, were not able to reach it [130]: while the importance of the three point system mechanism was stressed, options about proper pad placement on the thoracic convexity were divided 50% for the pad reaching or involving the apical vertebra and 50% for the pad acting caudal to the apical vertebra. There was agreement about the direction of the vector force, 85% selecting a 'dorso lateral to ventro medial' direction, but not about the shape of the pad to produce such a force. Principles related to three-dimensional correction achieved high consensus (80%-85%), but suggested methods of correction were quite diverse. This situation is reflected in the different corrective systems used throughout the world.

Looking at studies comparing different braces, we have already reported some studies:

• an RCT [134], that found a TLSO more effective than SpineCor;

• one meta-analysis [145], that was in favor of the Milwaukee brace, with Charleston being the less efficacious;

• one systematic review [141], that found the following pooled surgery rates: Boston Brace 12-17%; various braces (Boston-Charleston-TLSOs) 27-41; nigh time braces (Providence or Charleston braces) 17-25%; TLSO or Rosenberg brace 25-33; Wilmington 19-30%;

• two retrospective studies: one [138] obtained the best results with the Providence night time orthosis over a TLSO, the other [139] reported equal results with a rigid TLSO and SpineCor;

Reviewing the literature we also found:

• Among the oldest studies, Bunnell [146] reported similar results with a TLSO and Milwaukee brace in a preliminary retrospective study, while Montgomery [147] found that the Boston Brace was more successful than the Milwaukee brace irrespective of initial curve magnitude and skeletal maturity

• Katz [148] compared the Boston Brace to the Charleston bending brace. The first was more effective than the second, both in preventing curve progression and in avoiding the need for surgery. These findings were most notable for patients with curves of 36° to 45°, in whom 83% of those treated with a Charleston brace had curve progression of more than 5 degrees, compared with 43% of those treated with the Boston Brace.

• Howard [149] presented a retrospective cohort study on 170 patients who completed brace treatment: Forty-five patients with TLSO showed a mean progression of the curve of 1.1 degrees, 95 with Charleston worsened 6.5 degrees, and 35 with Milwaukee 6.3 degrees. Proportion of patients with more than 10 degrees of curve progression was 14% with TLSO, 28% with Charleston, and 43% with Milwaukee brace while those who underwent surgery were 18%, 31%, and 23% respectively.

• Weiss [79] performed a comparison of the survival rates of the Cheneau versus SpineCor with respect to curve progression and duration of treatment during pubertal growth spurt in two cohorts of patients followed up prospectively. At 24 months of treatment, 73% of the patients with a Cheneau brace and 33% of the patients with the SpineCor where still under treatment with their original brace; at 42 months the same percentages were 80% and 8% respectively.

• Yrjonen [150] studied retrospectively the Providence nighttime used by 36 lumbar and thoracolumbar scoliosis consecutive female patients with less than 35 degrees: progression of the curve > 5 degrees occurred in 27%, versus 36 matched patients treated with the Boston full-time that progression in 22% of cases.

• Negrini [151] compared the classical Lyon brace to the newly developed Sforzesco brace, based on the SPoRT concept (Symmetric, Patient-oriented, Rigid, Three-dimensional, active) with prospective, matched pairs controlled study. All radiographic and clinical parameters decreased significantly with treatment in both groups, apart from thoracic Cobb degrees with the Lyon brace. The Sforzesco brace had better results than the Lyon brace radiographically, for sagittal profile, aesthetics, and patient recovery (12 improved and 3 unchanged vs 8 and 5).

• Negrini [112] also studied a prospective cohort who had refused surgery treated with the Sforzesco brace to a Risser cast retrospective control group. Results were comparable between the two groups, with only minor differences in terms of scoliosis correction. On the contrary, straightening of the spine (decrease of the sagittal physiological curves) was much higher with the cast, while it was not clinically significant with the brace.

All these studies are not directly comparable, and the learning curve of the different systems can sometimes play a role in explaining the results. Moreover, in comparative studies the specific competence in making a specific brace can play a major role [135]: in this respect, even if it is not considered a good standard, comparison with historical controls treated with braces used before by the same treating team can offer good insights [112,138,139,150,151]. Today it is not possible to state with any certainty which brace is better than the other, and this is one of the reasons that drove the official publication of SOSORT to develop the Brace Thematic Series [152], where the different concepts are presented to allow a good comparison and a greater understanding of these treatment instruments [153-155]. Nevertheless it is already possible to see some trends:

• new alternative concepts have been developed trying to substitute the most invasive braces: this was true some years ago for TLSOs instead of Milwaukee, more recently for night time bending braces or SpineCor instead of TLSOs, and in the last years for the Sforzesco brace instead of casting; not all these new concepts have been able to prove their efficacy.

• in the meantime there is a struggle (mainly inside SOSORT) to progressively refine and strengthen some old concepts, like the Cheneau, Boston or Lyon braces, but also newly developed ones, like the Sforzesco and SpineCor.

In summation, examining all these studies in adolescent patients, it is clearly evident that something beyond the instrument (brace) plays a role in final results. These factors can include dosage, quality of bracing, compliance to treatment [156-158], family history, type of scoliosis and even a geographical distribution, but also team approach [114], that we will briefly review below.

##### Dosage, compliance and quality of bracing

Looking for dosage effect, Dolan did not find differences among the groups 16-18 hours (19-34% surgery rate), 18-23 hours (21-26%) and night time (17-25%) [141]; on the contrary, the meta-analysis by Rowe [145] reported that the twenty-three-hour regimens were significantly more successful than any other treatment, while the difference between the eight and sixteen-hour regimens was not significant. More recently, while Allington [159] reported no differences between full-time and part-time brace prescription both in curves below 30° and between 30° and 40°, Katz [160] has been able to check the real use of the brace by the patient through an heat sensor. A logistic regression analyses showed a "dose-response" curve in which the greater number of hours of brace wear correlated with lack of curve progression. Curves did not progress in 82% of patients who wore the brace more than twelve hours per day, compared with only 31% of those who wore the brace fewer than seven hours per day. As a result, dosage can be considered a possible major factor in explaining some of the results of bracing: in fact it has been shown that the more hours of daily brace weaning, the more the deformity comes back from the maximal correction ("concertina effect") [161].

Adherence to treatment is the second main issue to be considered. Many studies have underlined that referred compliance is correlated with final results [156,157,162]; compliance to bracing has been correlated to Quality of Life and psychological issues [163-166], even if patients declare that they would adhere to treatment provided its efficacy is proven [167]. Since patients during clinical evaluations overstate their adherence to treatment [168], heat sensors have been developed to check real compliance: it has been confirmed that both reported and estimated hours of brace wearing are inaccurate [169-174], and found that compliance is not correlated with the hours of bracing prescribed [173]. Night time wear is more accepted than daytime [175] and a "dose-response" to bracing seems to be confirmed [160,176]. It has also been proposed that it is possible to develop a progression model in single patients with a formula including the risk of progression at the beginning of brace treatment, plus the use in terms of brace tightness and wear time [177]. Nevertheless, compliance issues should be regarded from a wider angle than what usually reported, i.e. that, since patients are not compliant, bracing is not effective. SOSORT propose that compliance should be considered in terms of management of patients: in this perspective adherence to treatment is a characteristic neither of the treatment only, nor of the patient alone, but of the good interaction between these two factors, based on the active approach by an expert treatment team able to reduce the burden of the brace and increase the coping abilities of the patient [114,178]. Mainly for these reasons, SOSORT proposed its Recommendations [114].

Finally, the important factor quality of bracing. There is quite an agreement to judge it according to the in-brace correction [156-158,179-184], even if percentages reported in the literature as prognostic factors of final good results are quite variable from a minimum of 20-25% to 40-50% [156,157,185]. In-brace correction has become on one side the starting point to develop new braces [67,68,113,186-190], on the other a biomechanical reference for various studies [183,191,192]: recently a finite element model study confirmed the importance of immediate in-brace correction to predict long-term outcome of bracing treatment [183]. Other factors such as the absolute reduction of the Cobb angle (i.e., in rigid curves over 50 degrees) or 3D correction might also be important and should be considered in the future [180]: in fact, it is still possible that a great in-brace reduction corresponds to a worsening of other parameters, e.g. in the sagittal plane, finally driving to a flat-back and worse functional results [112]. In this respect, it is mandatory not to confuse the in-brace correction with the success of an orthotic treatment: while in-brace correction studies should be considered preliminary, only results at the end of treatment and/or at minimum of 1-2 years post treatment follow-up should be regarded as proves of efficacy. In any case, according to the actual knowledge in-brace correction should be regarded as the way to individually judge the quality of the brace applied to single patients.

All the criteria for inclusion, exclusion and outcome hava some drawbacks; one main problem is the fact that even the noncompliant patients are to be included in the studies and it seems that this is one of the criteria that is most frequently "forgotten". In this situation it is extremely difficult to compare two different studies and often the professional trying to offer the best treatment for his patients has the difficult task of comparing "apples with oranges". Apart from the inclusion and exclusion criteria as well as the assessment of brace effectiveness proposed by the SRS Committee, a few more guidelines for future studies should be proposed. All patients that accepted the treatment in a given time period should be included in the study regardless of their compliance. Patients that withdrawn from the treatment (changed the type of treatment, had surgery recommendation, etc.), regardless of their outcome, should be considered as failure of that specific treatment. All the patients that accepted a specific treatment should be followed up for at least 1-2 years after the completion of treatment and measurements should be taken at the beginning of the treatment, at the weaning point and at follow-up.

##### Efficacy in other populations

Adolescent idiopathic scoliosis with curves below 40-45° and still growing is the main field of brace treatment [141], but it has been applied as well in other populations, that we will briefly review here.

In juvenile idiopathic scoliosis, historically the percentages of surgery after treatment with braces ranged widely, with Tolo [193] reporting 27.2%, Figueiredo [194] 62%, Mannherz [195] 80%, McMaster [196] 86% and Kahanovitz [197] 100%. This clearly correlates with the difficulty in this specific population, where the expected progression rate could range between 70 and 95% [102]. More recently Coillard [102] reported that, with the SpineCor brace, out of 67 patients with a definite outcome, 32.9% corrected their Cobb angle by at least 5° and 10.5% had a stabilization of their Cobb angle, while 37.3% of patients where recommended for surgery before the authorized end of treatment (before skeletal maturity). Results depended on the amplitude of the Cobb angle: 26.3% of the patients with curves under 25 degrees eventually needed surgery while 51.8% of the second group (> 25°) had surgery recommended. Finally, Fusco [198] found a percentage of 9% of juvenile patients treated conservatively who finished treatment over 45°.

Also in infantile idiopathic scoliosis reported results are quite variable, as well as the treatment applied: serial casting is the most advocated [111,199-202], but also bracing alone has been used [199-201,203], mainly the Milwaukee brace [201,203]. The few case series reported generally include few patients with variable results, from a 100% surgery rate [204], to around 50% [199] or much less [201,205] (mainly if casts are used [199]). Mehta reported the widest case series of 136 children followed up for nine years: 94 children, referred and treated in the early stages (mean age 19 months-6 to 48, Cobb angle 32°-11° to 65°), resolved the deformity by a mean age of three years and six months, with no need of further treatment; 42 children, referred late (mean age 30 months-11 to 48, Cobb angle 52°-23° to 92°), reduced but not reversed scoliosis; 15 children (35.7%) were fused. The hypothesis of the author is that scoliosis can be reversed by harnessing the vigorous growth of the infant to early treatment by serial corrective plaster jackets [111].

Like in the adolescent type, puberty is the worst period also for infantile scoliosis, when surgery is mostly required [201]; single thoracic curves seem to have the worst outcomes when compared to double structural ones [203]; it has also been reported that best results are obtained in progressive types if treatment is started when the angulation is still under 30 degrees [205], or 60° and younger age [202], again mainly with casting [199,202]. When scoliosis is resolved or stabilized nonoperatively at an acceptable Cobb angle also normal cosmesis and pulmonary function is obtained; apparently this is not true if surgery is performed [200].

Finally, two papers recently focused on other groups:

• scoliosis over 45° who refused to be operated [77]. Out of 28 patients (curve range 45-58° Cobb) who reached the end of treatment (brace and exercises for 4.5 years) two patients (7%) remained above 50° but six patients (21%) finished between 30° and 35° and 12 patients (43%) finished between 36° and 40° Cobb. Improvements have been found in 71% of patients and a 5° Cobb progression in one patient.

• scoliosis of Risser 4-5 up to 20 years of age [206] (residual growth was 0.9 cm). Out of 23 patients requiring treatment or for esthetic reasons, or to try to reduce the deformity, curve improvements were found in 48% and decrease of the Esthetic Index in 30%.

##### Team role in bracing

SOSORT already produced a set of Recommendations in the paper "Standards of management of idiopathic scoliosis with corrective braces in everyday clinics and in clinical research" [114], grouped in 6 Domains: Experience/competence, Behaviours, Prescription, Construction, Brace Check, Follow-up. These recommendations, integrally reported below, constitute part of these Guidelines.

###### Recommendation 1 (Experience-competence)

The MD responsible for the treatment has to be experienced and should fulfill all these requirements:

1. training by a previous master (i.e. MD with at least 5 years of experience in bracing) for at least 2 years

2. at least 2 years of continuous practice in scoliosis bracing

3. prescription of at least 1 brace per working week (~45 per year) over the last 2 years

4. evaluation of at least 4 scoliosis patients per working week (~150 per year) over the last 2 years

Due to the actual situation of conservative treatment in many countries, this must be considered the ideal to be reached as soon as possible through education. Nevertheless, it must be recognized that experience and preparation is the only way to avoid problems to patients and reach adequate results in this field.

###### Recommendation 2 (Experience-competence)

The CPO constructing braces has to be experienced and should fulfill all these requirements

1. working continuously with a master MD (i.e. a MD fulfilling to recommendation 1 criteria) for at least 2 years

2. at least 2 years of continuous practice in scoliosis bracing

3. construction of at least 2 braces per working week (~100 per year) in the last 2 years

Due to the actual situation of conservative treatment in many countries, this must be considered the ideal to be reached as soon as possible through education. Nevertheless, it must be recognized that experience and preparation is the only way to avoid problems to patients and reach adequate results in this field.

###### Recommendation 3 (Behaviors)

To ensure optimum results, the MD, CPO and physiotherapist (PT) must work together as an interprofessional team. This can be accomplished, even if they are not currently located in the same workplace, through continuous exchange of information, team meetings, and verification of braces in front of single patients.

###### Recommendation 4 (Behaviors)

Commitment, time and counseling to increase compliance: MDs, CPOs and PTs have to give thorough advice and counseling to each single patient and family each time it is needed (at each contact for MDs and CPOs) provided they give as a team the same messages previously agreed upon.

###### Recommendation 5 (Behaviors)

All the phases of brace construction have to be followed for each single brace

1. prescription by a well trained and experienced MD (fulfilling recommendation 1 criteria)

2. construction by a well trained and experienced CPO (fulfilling recommendation 2 criteria)

3. checked by the MD in cooperation with the CPO, and possibly the PT

4. correction by the CPO according to MD indications

5. follow-up by the CPO, MD and PT.

###### Recommendation 6 (Prescription)

In each single prescription of a brace (case by case), the MD must:

1. write the details of brace construction (where to push and where to leave space, how to act on the trunk to obtain results on the spine) when not already defined "a priori" with the CPO

2. prescribe the exact number of hours of brace wearing

3. be totally convinced of the brace proposed and committed to the treatment

4. use any ethical means to increase patient compliance, including thorough explanation of the treatment, aids such as photos, brochures, video, etc.

###### Recommendation 7 (Construction)

In each single construction of a brace, case by case, the CPO has to:

1. check the prescription and its details and eventually discuss them with the prescribing MD, if needed, before construction

2. fully execute the agreed prescription

3. be totally convinced of the brace proposed and committed to the treatment

4. use any ethical means to increase patient compliance, including thorough explanation of the treatment, aids such as photos, brochures, video, etc.

###### Recommendation 8 (Brace Check)

In each single check of a brace, case by case, the responsible MD in partnership with the CPO has to:

1. verify accurately if it fits properly and fulfils the needs of the individual patient

2. check the scoliosis correction in all three planes (frontal, sagittal and horizontal)

3. check clinically the esthetic correction

4. maximize brace tolerability (reduce visibility and allow movements and activity of daily life as much as possible for the chosen technique)

5. apply all changes needed and, if necessary, even rebuild the brace without extra-charge for patients

6. check the corrections applied

7. check that the patient (and/or his/her parents) is able to apply or put on the brace properly

8. access the patient's mood and counsel with the family at brace delivery and at other follow-ups.

###### Recommendation 9 (Brace Check)

The check of each single brace must be a clinical and/or radiographic check.

###### Recommendation 10 (Follow-up)

The MD, CPO and PT must check the brace and patient compliance regularly (MDs and CPOs each time they see the patient), and reinforce the usefulness of brace treatment to the patient and his/her family.

###### Recommendation 11 (Follow-up)

The MD has to follow-up the braced patient regularly, at least every 3 to 6 months. Standard intervals have to be reduced according to individual needs (first brace, growth spurt, progressive or atypical curve, poor compliance, request of other team members-CPO, PT ...). Using tools (written protocols, recalls, etc.) to keep patients informed of their follow-up is strongly suggested.

###### Recommendation 12 (Follow-up)

The brace has to be changed for a new one as soon as the child grows or the brace loses efficacy, and this need can be suggested by the CPO, but is the responsibility of the treating MD.

###### Recommendation 13 (Follow-up)

The CPO has to regularly check the brace. To avoid any problems, he/she has to refer to the treating MD.

###### Recommendation 14 (Follow-up)

The PT has to check the brace regularly. To avoid any problems, she/he has to refer to the treating MD. As a member of the treating team, he/she has to be trained to face the problems of compliance, or the needs for more explanation by the patient or his/her family. In case she/he is not entirely a member of the treating team the PT must not act autonomously and must refer to the treating MD.

##### Other issues

It is not possible in this review of the literature to fully consider the complex and currently debated topics like:

• CAD-CAM versus plaster molding in brace construction: research is reaching the conclusion that the way in which the brace is constructed does not interfere with final results, nor with patients' sensations [180,187,189,207];

• finite element modeling of brace efficacy: models are showing the efficacy of bracing in reducing spinal load and applying corrective moments to the spine; moreover they are helping in refining brace construction, but there is still a long way to go [183,192,208-210];

• 3D classifications and their effect on brace construction and results' evaluation: some more years are needed to reach the first clinically useful applications [65,69-72,211].

These topics, and others that research will produce in the next years, will be reviewed and considered in depth in next Editions of the SOSORT Guidelines.

#### Recommendations on "Bracing"

1. Bracing is recommended to treat adolescent idiopathic scoliosis (SoR: B) (SoE: III) [76,78,131,132,137-139]

2. Bracing is recommended to treat juvenile and infantile idiopathic scoliosis as the first step in an attempt to avoid or at least postpone surgery to a more adequate age (SoR: B) (SoE: IV) [102,193,194,198-201,203]

3. Casting is recommended to treat infantile idiopathic scoliosis to try stabilizing the curve (SoR: B) (SoE: IV) [111,199-202]

4. It is recommended not to apply bracing to treat patients with curves below 15 ± 5° Cobb, unless otherwise justified in the opinion of a clinician specialized in conservative treatment of spinal deformities (SoR: B) (SoE: VI)

5. Bracing is recommended to treat patients with curves above 20 ± 5° Cobb, still growing, and demonstrated progression of deformity or elevated risk of worsening, unless otherwise justified in the opinion of a clinician specialized in conservative treatment of spinal deformities (SoR: B) (SoE: III) [76,78,131,132,137-139,141]

6. It is recommended that each treating team provide the brace that they know best and are most prepared to manage: due to the actual knowledge, there is no brace that can be recommended over the others (SoR: C) (SoE: IV) [134,138,139,141,145]

7. It is recommended that braces are worn full time or no less than 18 hours per day at the beginning of treatment, unless otherwise justified in the opinion of a clinician specialized in conservative treatment of spinal deformities (SoR: B) (SoE: IV) [145,160]

8. Since there is a "dose-response" to treatment, it is recommended that the hours of bracing per day are in proportion with the severity of deformity, the age of the patient, the stage, aim and overall results of treatment, and the achievable compliance (SoR: B) (SoE: IV) [145,160]

9. It is recommended that braces are worn until the end of vertebral bone growth and then the wearing time is gradually reduced, unless otherwise justified in the opinion of a clinician specialized in conservative treatment of spinal deformities (SoR: B) (SoE: V)

10. It is recommended that the wearing time of the brace is gradually reduced, while performing stabilizing exercises, to allow adaptation of the postural system and maintain results (SoR: B) (SoE: IV) [91,142-144,212]

11. It is recommended that any mean is used to increase and monitor compliance, including heat sensors and a careful adherence to the recommendations defined in the SOSORT Guidelines for Bracing Management (SoR: B) (SoE: VI) [114,169-174]

12. It is recommended that quality of the brace is checked through an in-brace x-ray (SoR: B) (SoE: IV) [156-158,179-184]

13. It is recommended that the prescribing physician and the constructing orthotist are experts according to the criteria defined in the SOSORT Guidelines for Bracing Management (SoR: B) (SoE: V) [114]

14. It is recommended that bracing is applied by a well trained therapeutic team, including a physician, an orthotist and a therapist, according to the criteria defined in the SOSORT Guidelines for Bracing Management (SoR: B) (SoE: V) [114]

15. It is recommended that all the phases of brace construction (prescription, construction, check, correction, follow-up) are carefully followed for each single brace according to the criteria defined in the SOSORT Guidelines for Bracing Management (SoR: A) (SoE: V) [114]

16. It is recommended that the brace is specifically designed for the type of the curve to be treated (SoR: A) (SoE: V)

17. It is recommended that the brace proposed for treating a scoliotic deformity on the frontal and horizontal planes should take into account the sagittal plane as much as possible (SoR: A) (SoE: V)

18. It is recommended to use the least invasive brace in relation to the clinical situation, provided the same effectiveness, to reduce the psychological impact and to ensure better patient compliance (SoR: B) (SoE: V)

19. It is recommended that braces do not so restrict thorax excursion in a way that reduces respiratory function (SoR: A) (SoE: V)

20. It is recommended that braces are prescribed, constructed and fitted in an out-patient setting (SoR: B) (SoE: VI)

## Conservative treatments other than bracing

### Physiotherapeutic Specific Exercises to prevent scoliosis progression during growth

#### Methods

In February 2011 we performed a search of Medline from its inception, with no language limitations. We used the terms *("Exercise Therapy"[Mesh]) AND "Scoliosis"[Mesh] *and we found 206 papers; after reviewing the titles, 66 were considered of interest; looking at the abstracts 41 were selected and retrieved in full text. We also searched: the abstracts of all SOSORT Meetings, from the first one in 2003 to 2010; the personal files and knowledge of all the authors; the papers retrieved with all the other searches listed in these Guidelines; the references sections of all retrieved papers. The selection criteria used in all these searches were: pertinence for the topic "Physiotherapeutic Specific Exercises to prevent scoliosis progression"; presence of the abstract; numerical results in relation to scoliosis; retrievability in full text; all languages.

#### Results

SOSORT has published in Scoliosis Journal a Consensus Paper titled "Physical Exercises in the Treatment of Idiopathic Scoliosis at Risk of brace treatment-SOSORT Consensus paper 2005" [213]: this can serve as reference for specific insights. In this Consensus some characteristics of Physiotherapeutic Specific Exercises have clearly been stated with almost unanimity among SOSORT experts: auto-correction in 3D, training in ADL, stabilizing the corrected posture, and patient education should be always included.

Moreover, a Cochrane review on exercises that follows the protocol presented in 2009 [214], has been submitted and it is now under review: this review found 2 papers of high interest, one RCT that provided low quality evidence in favor of exercises used together with other treatments [215], and one cohort observational prospective trial with a concurrent control group that gave very low quality evidence in favor of specific versus general exercises to avoid brace prescription [216].

In the orthopaedic literature prevails the so-called "exercise dogma" [217,218], that states that exercises are not useful for scoliosis treatment; this is widespread [48,219,220], and presumably comes from an old paper published down in 1979 [221], the only one against the effectiveness of Physiotherapeutic Specific Exercises. Consequently, the old systematic reviews concluded on the inefficacy of exercises [222]; more recently, three comprehensive systematic reviews published in last years by the same group [223-225], and to a lesser extension another one [226,227], have exhaustively evaluated studies on the efficacy of specific exercise programs in reducing the probability of progression of idiopathic scoliosis. These reviews found that the general methodology used in studies published so far has generally been of poor quality, even though, except for 1 study (the oldest one) [221], all study results indicate that treatment is useful [215,216,228-244]. The authors of these systematic reviews concluded that, as far as we know today, Physiotherapeutic Specific Exercises may be proposed to patients.

The exercises papers have been tentatively classified according to the auto-correction proposed [225]: extrinsic (maximal correction obtained also with the help of gravity, positioning devices and/or limbs placement) [228,235-239,242-244], intrinsic (maximal correction achievable without any external aids) [216,229,230,232,234], no auto-correction but asymmetric exercises [215,240,241], no auto-correction and symmetric exercises [221,231,233]. According to these reviews, until now the Physiotherapeutic Specific Exercises School with some published proves of efficacy (in alphabetical order) include: DoboMed [235], Lyon [229,230,234], MedX [240,241], Schroth (either as Scoliosis Intensive Rehabilitation [228,237,242,245], or outpatient approach [238,244]), SEAS [216,232], side shift [236,239,243].

A major drawback, however, is the unevenness of information about the natural history of progression of scoliosis [129,246]. The probability that the curve will worsen depends on patient age at diagnosis, type and severity of curve, sex and skeletal maturity [129,247,248]. From 25% to 75% of curves found at screening may remain unchanged, whereas from 3% to 12% of curves may improve [35,129]. Treatment decisions should be individualized, considering the probability of curve progression, based on curve magnitude, skeletal maturity, patient age and sexual maturity [48,249].

Finally, we have to consider also the concept of acceptability of treatment together with efficacy and effectiveness: when facing a progression risk of 25%, families preferred the use of Physiotherapeutic Specific Exercises for prevention instead of awaiting a possible progression of the deformity to be later treated with a brace [250].

#### Recommendations on "Physiotherapeutic Specific Exercises to prevent scoliosis progression during growth"

21. Physiotherapeutic Specific Exercises are recommended as the first step to treat idiopathic scoliosis to prevent/limit progression of the deformity and bracing (SoR: B) (SoE: II) [214,215,223-225]

22. It is recommended that Physiotherapeutic Specific Exercises follow SOSORT Consensus and are based on auto-correction in 3D, training in ADL, stabilizing the corrected posture, and patient education (SoR: B) (SoE: VI) [213]

23. It is recommended that Physiotherapeutic Specific Exercises follow one of the School that have shown the effectiveness of their approach with scientific studies (SoR: B) (SoE: III) [216,228-230,232,234-244]

24. It is recommended that Physiotherapeutic Specific Exercise programs are designed by therapists specifically trained in the School they use (SoR: B) (SoE: VI)

25. It is recommended that Physiotherapeutic Specific Exercises are proposed by therapists included in scoliosis treatment teams, with close cooperation between all members (SoR: B) (SoE: V) [114]

26. It is recommended that Physiotherapeutic Specific Exercises are individualized according to patients needs, curve pattern, and treatment phase (SoR: B) (SoE: III) [216,228-230,232,234-244]

27. It is recommended that Physiotherapeutic Specific Exercises are always individualized even if performed in small groups (SoR: B) (SoE: VI)

28. It is recommended that Physiotherapeutic Specific Exercises are performed regularly throughout treatment to achieve best results (SoR: B) (SoE: VI)

### Physiotherapeutic Specific Exercises during brace treatment and surgical therapy

#### Methods

In February 2011 we performed a search of Medline from its inception, with no language limitations. For this section we used the terms *("Exercise Therapy"[Mesh]) AND "Scoliosis"[Mesh] *and *"Braces"[Mesh] AND "Scoliosis"[Mesh] AND (hasabstract[text] AND (Clinical Trial[ptyp] OR Meta-Analysis[ptyp] OR Practice Guideline[ptyp] OR Randomized Controlled Trial[ptyp] OR Review[ptyp])) *outlined above; we also add a specific search with the terms *(("Scoliosis/surgery"[Mesh]) AND "Scoliosis/rehabilitation"[Mesh]) OR (("Scoliosis/surgery"[Mesh]) AND "Exercise Therapy"[Mesh])*. We also searched: the abstracts of all SOSORT Meetings, from the first one in 2003 to 2010; the personal files and knowledge of all the authors; the papers retrieved with all the other searches listed in these Guidelines; the references sections of all retrieved papers. We finally retrieved 40 relevant papers. The selection criteria used in all these searches were: pertinence for the topic "Physiotherapeutic Specific Exercises during brace treatment and surgical therapy"; presence of the abstract; numerical results in relation to scoliosis; retrievability in full text; all languages.

#### Results

Even if in the past Physiotherapeutic Specific Exercises to be performed as a companion of brace treatment have been proposed by most of the authors who developed specific braces, such as for the Milwaukee [251-253], Boston [254], Lyon [255,256] and Chêneau braces [257-259], this part of conservative scoliosis treatment seems to have been neglected as well [260]. Nevertheless, recently Physiotherapeutic Specific Exercises, beyond the original ones, have been associated to classical braces, like side-shift for the Milwaukee brace [143,261,262], or Schroth for the Chêneau [144,179,263-265]; moreover, the newly developed Sforzesco brace is born strictly associated with exercise performance [77,91,266].

When compared to a systematic review of cohort studies on bracing that formally excluded all protocols with exercises [141], all studies combining the two treatments showed very good results [114]: surgery rate dropped from the average of 22% (observed) or 23% (treated) [141] to 0-7% in the efficacy analysis [78,91,142-144,267], or 10-14% in the worst case analysis [91,142]. This was true independently by the brace used: Milwaukee and side-shift [143], Chêneau and Schroth [142,144,268], cast or Lyon or Sibilla and SEAS [78,91]. The only exception to this rule is a recently published paper in which exercises have not been used, that reported a 0% surgery rate according to the SRS criteria [76]; in this study SOSORT criteria [114] have been utilized: this opens up the possibility that, beyond the specific effect of exercises, the physical therapist's approach can have a fundamental role in maintaining compliance as proposed by the SOSORT Guidelines for Brace Treatment Management [114]. Another main point in this study that may have improved the compliance is that the patients were all managed by the same physician.

Recently, one paper winning the SOSORT Award has shown the importance of exercises in reducing the loss of correction in the brace weaning phase [212]; another study demonstrated some usefulness of preparation to brace exercises [233]. In this respect, an old controlled randomized study on a small population showed that in adolescents wearing a brace, exercises are more effective than traction in improving curvature on lateral bending (i.e. increasing mobility, that should help brace action) [269]. Historically it has been shown that thoracic flexion exercises are immediately effective in reducing the vertebral rotation and lateral deviation in Milwaukee brace [270]; but in a prospective study, no significant differences have been found between 12 compliant and 12 noncompliant patients with primary right thoracic idiopathic scoliosis treated with trunk muscles strengthening exercises and Milwaukee brace [271].

The neurophysiological basis of an integration of bracing and exercises in a complete rehabilitation program for adolescent idiopathic scoliosis has been described [272]. Most of the Schools used the same exercises during brace treatment proposed without the orthosis, even if the Lyon [256,273] and SEAS [94,212,233] ones propose specific preparatory and in-brace exercises, different from those usually performed without the brace.

Finally, exercises and surgical treatment. They have been advocated as an important part of the rehabilitation process following fusion [16,256,274], nevertheless the Scoliosis Research Society surgeons, when inquired if they prescribed physical therapy at hospital discharge, answered that it was unlikely [275]. It has been reported as painful to patients 10 or more years after scoliosis surgery a highly significant pain and pain frequency reduction through a multimodal treatment including stabilizing postural and respiratory exercises lasting several hours a day (5 1/2 to 7 hours) [276].

#### Recommendations on "Physiotherapeutic Specific Exercises during brace treatment and surgical therapy"

29. It is recommended that Physiotherapeutic Specific Exercises are performed during brace treatment (SoR: B) (SoE: III) [78,91,142-144,267]

30. It is recommended that, while treating with Physiotherapeutic Specific Exercises, therapists work to increase compliance of the patient to brace treatment (SoR: B) (SoE: V) [114]

31. It is recommended that spinal mobilization Physiotherapeutic Specific Exercises are used in preparation to bracing (SoR: B) (SoE: II) [233,269]

32. It is recommended that stabilization Physiotherapeutic Specific Exercises in autocorrection are used during brace weaning period (SoR: B) (SoE: IV) [212]

33. It is recommended that Physiotherapeutic Specific Exercises in painful operated patients are used to reduce pain and increase function (SoR: B) (SoE: IV) [276]

### Other conservative treatments

#### Methods

In February 2011 we performed a search of Medline from its inception, with no language limitations. We used the terms *((((("Musculoskeletal Manipulations"[Mesh])) OR "Homeopathy"[Mesh]) OR "Acupuncture"[Mesh]) OR "Diet"[Mesh]) AND "Scoliosis"[Mesh] *and we found 68 papers; after reviewing the titles, 13 were considered of interest; looking at the abstracts 7 were maintained and retrieved in full text. We also searched: the abstracts of all SOSORT Meetings, from the first one in 2003 to 2010; the personal files and knowledge of all the authors; the papers retrieved with all the other searches listed in these Guidelines; the references sections of all retrieved papers. The selection criteria used in all these searches were: pertinence for the topic "Other conservative treatments"; presence of the abstract; numerical results in relation to scoliosis; retrievability in full text; all languages.

#### Results

When looking at other conservative approaches beyond Physiotherapeutic Specific Exercises, some case reports of improvement of scoliosis with mobilisation techniques applied as a stand-alone treatment have been reported in the short- (weeks) [277] and medium-term (months) [278]; the same has been done on mobilization together with other stabilising techniques in the medium- [279] and long-term (years) on spinal curve [280] and chest expansion [281]; a short-term case series has been reported as well [282]. Nevertheless, a systematic review was not able to conclude the effectiveness of manual treatment due to the lack of good studies [283]. Finally, there are no scientific studies on the therapeutic efficacy of shoe inserts (excluding heel lifts lifts), conventional and homeopathic medicines, acupuncture or specific dietary regimens for the correction of idiopathic scoliosis in adolescence.

#### Recommendations on "Other conservative treatments"

34. It is recommended that manual therapy (gentle, short-term mobilization, or releasing soft tissues techniques) is proposed only if associated with stabilization Physiotherapeutic Specific Exercises (SoR: B) (SoE: V) [283]

35. It is recommended that correction of real leg length discrepancy, if needed, is decided by a clinician specialized in conservative treatment of spinal deformities (SoR: B) (SoE: VI)

36. It is recommended that shoe inserts (excluding heel lifts), conventional and homeopathic medicines, acupuncture, or specific dietary regimens are not used to correct a spinal deformity (SoR: B) (SoE: VI)

### Respiratory function and exercises

#### Methods

In February 2011 we performed a search of Medline from its inception, with no language limitations. We used the terms *("Respiration"[Mesh]) AND "Scoliosis"[Mesh] *and we found 182 papers; after reviewing the titles, 42 were considered of interest; looking at the abstracts 35 were maintained and retrieved in full text. We also searched: the abstracts of all SOSORT Meetings, from the first one in 2003 to 2010; the personal files and knowledge of all the authors; the papers retrieved with all the other searches listed in these Guidelines; the references sections of all retrieved papers. The selection criteria used in all these searches were: pertinence for the topic "Respiratory exercises"; presence of the abstract; numerical results in relation to scoliosis; retrievability in full text; all languages.

#### Results

A series of studies mainly in adolescents with scoliosis between 30 and 60° have demonstrated various types of respiratory impairments in patients: abnormal ventilation patterns, mainly restrictive [284-286]; impaired function of respiratory muscles [284,286]; restriction [285,287] and asymmetric motion of the chest wall, with localized alterations [288]; abnormal patterns of ventilation during exercise [289], similar to that seen in patients with severe COPD [290]. Among the possible causes, the deformity plays a role in terms of lateral flexion [284] (with some doubts [291]), vertebral rotation [292,293] and stiffness [285]; the sagittal diameter [292], overall dimensions [291,292] and stiffness [285] of the thoracic cage are important as well [294,295]

Exercise capacity appears impaired as well [284,296-298], but without a direct correlation with ventilatory limitations or abnormality in lung volumes [284,297,298]: determining factors appear to be deconditioning and lack of regular aerobic exercise [297,298], as it can be shown also by lower limb muscle function [284] and also the severity of the scoliosis curve [296].

The natural history cohort followed-up 50 years by Weinstein seems to point to the conclusion that cardio-respiratory failure is not a common problem in the adult with adolescent idiopathic scoliosis [80], even if these results have been considered with some criticism, due to possible methodological flaws [49,299]. Pehrsson [300,301] showed that cardiorespiratory failure occurs only in cases of severe scoliosis that had its onset in pre-puberty and with a strong tendency to progression, wherein vital capacity was the strongest indicator for possible respiratory failure. An interesting study was performed in adults with infantile-onset scoliosis, showing a correlation among treatment performed and resulting pulmonary function: those whose scoliosis resolved or was stabilized by non-operative means had normal pulmonary function; those who were managed by casting or bracing and underwent surgery after age 10 had acceptable pulmonary function; but those whose deformity necessitated early surgery had recurrence of deformity and diminished respiratory function [200].

All these studies point to the importance of performing general aerobic activities (including sport) and respiratory training to improve exercise capacity and respiratory muscles functioning, while decreasing deconditioning and thoracic stiffness. Nevertheless doubts could be raised in terms of asymmetric stress due to increased respiratory effort [302], and some old studies showed bad results [303,304]. Also, the role of Physiotherapeutic Specific Exercises can be discussed: while SOSORT experts suggested the use of respiratory exercises and education [305], one paper showed in adult scoliosis patients an increase in vital capacity and in chest wall expansion that would allow treatment of associated restrictive ventilatory diseases [306]; another paper demonstrated improvements of electrocardiographic parameters of right-heart stress [307]. If scoliosis is of very high degree, nocturnal nasal intermittent positive pressure ventilation (together with long-term oxygen therapy) can have a positive effect improving exercise capacity [308], survival rate [309], health-related quality of life and decreasing the hospitalization rate [310].

Bracing can impact pulmonary function, even if results are contradictory [311-315]. In scoliosis girls wearing a Boston-type brace a two-month aerobic training sustained or improved significantly the parameters of pulmonary function, while they were reduced in the control group with no exercises in Milwaukee brace [316]. In most of the studies, correction and surgical stabilization of the curve lead to only a slight improvement of pulmonary function, with some exceptions.

#### Recommendations on "Respiratory function and exercises"

37. It is recommended that, when needed, exercises to improve respiratory function are used (SoR: B) (SoE: V)

38. It is recommended during brace treatment to use exercises to improve respiratory function (SoR: B) (SoE: IV) [316]

39. It is recommended the use of Physiotherapeutic Specific Exercises to train regional respiratory strategies to promote the expansion and ventilation of specific lung compartments (SoR: B) (SoE: IV) [306]

### Sports activities

#### Methods

In February 2011 we performed a search of Medline from its inception, with no language limitations. We used the terms *("Sports"[Mesh]) AND "Scoliosis"[Mesh] *and we found 105 papers; after reviewing the titles, 24 were considered of interest; looking at the abstracts 11 were maintained and retrieved in full text. We also searched: the abstracts of all SOSORT Meetings, from the first one in 2003 to 2010; the personal files and knowledge of all the authors; the papers retrieved with all the other searches listed in these Guidelines; the references sections of all retrieved papers. The selection criteria used in all these searches were: pertinence for the topic "Sports activities"; presence of the abstract; numerical results in relation to scoliosis; retrievability in full text; all languages.

#### Results

It has been suggested that general sports activities can be an active counterpart of Physiotherapeutic Specific Exercises [256]. Even if some confusion seems to remain in the literature between general sport activities and Physiotherapeutic Specific Exercises [317,318], their different role may be understood by looking at gross specific differences: Physiotherapeutic Specific Exercises are developed to purposely face scoliosis impairments and biomechanics [305], while the goal of sport activities is to either obtain agonistic results or improve fitness and wellness; moreover, Physiotherapeutic Specific Exercises work explicitly on the spinal muscles and posture control [217,272,305,319], while sports activities on the big muscles related with limb movements. Nevertheless an interaction and overlap between the two types of physical activities exists and can be recognized. In particular, the specific social and educational role of sports activities in terms of play, either at or outside school, should never be neglected, since patients with scoliosis should play "the same as and even more than others" [2]. It has been highlighted how psychological and social aspects are related to the patient's negative image of his or her own body [320]: physical activity allows patients to work on these aspects and to stay involved with their peer group, particularly but not only during physical education at school.

Participating in various types of sports activities doesn't seem to affect the presence or degree of scoliosis [317]. Scoliotic patients prefer to practice sports like gymnastics (usually started before discovering scoliosis) [321,322]: this seems to be linked to a higher prevalence of joint laxity than controls [322]. Delay in menarche and generalized joint laxity are common in rhythmic gymnastic trainees as well, and a 10-fold higher incidence of scoliosis was found in this group (12%) than in normal controls (1.1%) [323]: a "dangerous triad" has been hypothesized, including generalized joint laxity, delayed maturity, and asymmetric spinal loading. Similarly, an increased incidence of scoliosis has been reported in ballet dancers (24%) [324], and a separate etiology for ballet and rhythmic gymnastics than in adolescent idiopathic scoliosis has been hypothesized [325]. However, in a pair of 13.5-year-old female monozygotic twins who were high-level athletes in synchronized swimming, only one showed a 32 degrees thoracolumbar curve: this seems to suggest that factors other than genetics and sport activities play important roles [326].

Looking at other sports, even if swimming has been proposed traditionally as a good sport activity for scoliosis (and even prescribed by some physicians as a treatment), a 6.9% incidence of scoliosis, 3.5-fold that in normal controls, has been reported in swimmers [327]. There are no papers at all looking at asymmetric sports, traditionally blamed, but without any scientific evidence.

Adolescents with double major curves practice more sports activities than those with a single major curve, but both groups less than normal controls: it has been hypothesised that the first scoliosis group can be less subject to scoliosis-related biomechanical repercussions leading to a better balance control [321]. Over the long term, patients with important idiopathic scoliosis suffer impairment of their sports activities compared with age-matched controls, due to functional impairment and back pain. Sports activity is not more restricted after extended spinal fusion than it is after non-operative treatment [328]. In this respect, the Scoliosis Research Society surgeons return patients to noncontact sports between 6 months and 1 year post-operatively, while contact sports were generally withheld until 1 year after surgery; close to 20% of respondents required, and 35% suggested, that patients never return to collision sports. Twenty percent of surgeons reported having notable adverse outcomes attributed to athletic activity after surgery [275].

#### Recommendations on "Sports activities"

40. It is recommended that sports is not prescribed as a treatment for idiopathic scoliosis (SoR: C) (SoE: III) [317,321-324,326,327]

41. It is recommended that general sports activities are performed because of the specific benefits they offer to patients in terms of psychological, neuromotor and general organic well-being (SoR: B) (SoE: V)

42. It is recommended that, during all treatment phases, physical education at school is continued. Based on the severity of the curve and progression of the deformity and the opinion of a clinician specialized in conservative treatment of spinal deformities, restrictions may be placed on practicing certain types of sports activities (SoR: B) (SoE: V)

43. It is recommended that sports activities are continued also during brace treatment because of the physical (aerobic capacity) and psychological benefits these activities provide (SoR: B) (SoE: IV) [316]

44. It is recommended that, during brace treatment, contact or highly dynamic sport activities are performed with caution (SoR: B) (SoE: VI)

45. It is recommended that competitive activities that greatly mobilize the spine are avoided in patients with scoliosis at high risk of progression (SoR: C) (SoE: III) [284-287,317,322-324]

## Assessment

SOSORT has published in Scoliosis Journal a Consensus Paper titled "Methodology of evaluation of morphology of the spine and the trunk in idiopathic scoliosis and other spinal deformities-6th SOSORT consensus paper" [329]: this can serve as reference for specific insights.

Since scoliosis is diagnosed as idiopathic only by exclusion, it is mandatory at the first evaluation to collect family and personal clinical history and perform a full medical and neurological exam [329].

The main evaluation test in the clinical examination of patients with scoliosis is the Adam's forward bending test. A positive result to the test is pathognomic for scoliosis [330]. The test's positive predictive value varies since it is proportional to the degree of curvature and depends on operator experience [331].

The Scoliometer [332,333] measures the hump appearing as a consequence of the Adam's test: it is an evaluation tool that has proven highly useful. The Scoliometer measures the angle of trunk inclination (ATI, or ATR-Angle of Trunk Rotation) and has a high inter-observer reproducibility, which permits the determination of cut-off points above which a radiographic study is indicated. It has a sensitivity of about 100% and a specificity of about 47% when an ATI angle of 5° is chosen. At an ATI angle of 7° sensitivity drops to 83% but specificity rises to 86% [28,334,335]. While 7° can be considered a good cut-off in a surgical setting, when prevention is desired through a good conservative approach, 5° is a better cut-off.

Measurement of the hump is another instrument that can provide a further parameter of evaluation and differs from the Scoliometer in that it measures the height of the difference between curve concavity and convexity [89,336]. A cut-off point of 5 mm has been defined as significant for measuring back hump [336,337], and the reliability of this measurement has been reported [89,334]. A new instrument demonstrating high reproducibility has also been recently tested [338].

Being aesthetics a major concern for AIS patients [42], a specific assessment of trunk asymmetries should be used. The TRACE scale has been recently proposed and validated: it's a 12 point scale based on a visual assessment of shoulders, scapulae, waist and hemithorax asymmetries. Intra-rater repeatability was fair, being the minimum significant change three out of twelve, while inter-raters was poor being the minimum significant change four [88]. Also the self-evaluation by patients is very important in this respect, and validated scales like the Walter-Reed and TAPS have been proposed [339-342].

Quality of life (QoL) issues and disability are other main points to be considered in the treatment of IS patients [42]. A series of instruments (questionnaires) have been proposed in these years to evaluate QoL, starting for the first one that almost constitutes a standard, the SRS-22 [343-346]. Nevertheless, for clinical everyday conservative use the SRS-22 shows some limits, and other questionnaires have been developed like the BrQ [163,347-350] and the BSSQ [347,351-354].

The sagittal profile of the spine is frequently modified in scoliosis patients, and a sagittal measurement is recommended. Many different tools exist, like the plumbline, the Inclimed and the Arcometer [355-357].

Radiographic examination remains the reference standard: it is important to use one of the clinical cut-off points mentioned above (ATI or hump), before ordering a radiographic study, and during regular follow-up to reduce the burden of radiations [329]. Cobb angle measurements on the same radiographic image had an intra- and inter-observer variability of 3-5° and 6-7°, respectively [358]; this classically reported error increases when the postural, and even diurnal changes in different exams are considered [358,359]. Radiographic measurement of the vertebral rotation using Perdriolle's torsiometer has been shown to be reproducible [360]. Based on the same principle, use of Raimondi's tables or ruler makes measurement easier and slightly more reproducible [361].

In infantile idiopathic scoliosis frontal plane radiographs a very important measurement has been proposed by Mehta: the rib-vertebra angle, that provide a prognostic factor allowing the examiner to distinguish between evolving and resolving scoliosis [111,362,363].

The radiographic exam of the sagittal plane is important, but it has inherent difficulties due to the need to move the arm from the anatomical position to show the spine [357,364-366]: as a consequence, after performing it for diagnostic purposes, surface measurements can substitute it in follow-up of the patients [329,367,368].

The Risser sign [369] constitutes a further parameter for radiographic evaluation and is useful in indicating the patient's growth status, since Risser grading can be done using the same radiographic film to evaluate the scoliosis [128,370-372]. Other essential parameters to be considered are radiographic maturity of the ring apophyses (annular apophyses), appearance of menarche in girls, and Tanner staging [329]. Other diagnostic imaging procedures are in use in idiopathic scoliosis, like various radiographic technique beyond classical projections [373], MRI [373,374], neurophysiological exams [375]. Nevertheless, beyond their importance in the surgical setting, in the everyday use for conservative purposes, these techniques are not supported by the actual evidence, unless there are symptoms and signs of neurological compromise: only in these cases, in fact, a specific diagnosis is useful [376].

"Hot" topics of research that are almost ready to enter in the everyday clinical world and that will presumably be addressed in a few years with the next edition of these Guidelines include:

• Surface topography measurements, that have been widely used for research purposes in these years, but only recently are apparently entering the clinical everyday world [329,367,368]. Esthetics and sagittal plane evaluations could presumably become everyday clinics quite rapidly.

• Genetic evaluation [Ogilvie: 123-126]. Nevertheless, prudence is advised in using these tools to decide if to treat or not patients: in fact, moving from research, even if performed in wide samples of some hundreds of patients, to the general population requires caution.

Finally, a key point to be considered in the assessment of idiopathic scoliosis is screening: through an initial general surface measurement, and a subsequent selected clinical expert evaluation to eventually reach a final radiographic exam, the deformity can be detected early and treated to avoid progression. Even if doubts have been raised, screening for idiopathic scoliosis in asymptomatic adolescents is to be recommended [377]. SOSORT has published in Scoliosis Journal a Consensus Paper titled "SOSORT consensus paper: school screening for scoliosis: Where are we today?"[377]: this can serve as reference for specific insights.

### Recommendations

46. School screening programs are recommended for the early diagnosis of idiopathic scoliosis (SoR: B) (SoE: IV)

47. It is recommended that, every time they evaluate children aged from 8 to 15 years, pediatricians, general practitioners and sports physicians perform the Adam's test for scoliosis screening purposes, using the Scoliometer (SoR: A) (SoE: V)

48. It is recommended that the Adam's test use is spread in the school community and among all people that are engaged in the health of children (parents included) (SoR: B) (SoE: V)

49. It is recommended that diagnostic evaluation is carried out by clinicians specialized in spinal deformities (SoR: B) (SoE: IV)

50. It is recommended that patients are always examined by the same clinicians specialized in spinal deformities. In settings in which this is not possible, it is recommended regular standardization and validation processes of the methods used (SoR: B) (SoE: IV)

51. It is recommended for clinical follow-up the use of validated assessment methods and standard clinical data collection forms (SoR: A) (SoE: V)

52. It is recommended that the assessment include pathologic, cosmetic, psychological, functional and family aspects (SoR: B) (SoE: V)

53. It is recommended that the sagittal alignment of the spine is evaluated (SoR: A) (SoE: V)

54. It is recommended the Scoliometer and Humpmeter for clinical evaluation and follow-up of patients (SoR: B) (SoE: V)

55. It is recommended during growth that clinical follow-up examinations are performed at least twice a year, a part periods of rapid growth (pubertal spurt, first three years of life) (SoR: B) (SoE: V)

56. It is recommended not to perform x-rays if the Adam's test is negative and the Scoliometer value is below 5°, unless otherwise justified in the opinion of a clinician specialized in conservative treatment of spinal deformities (SoR: B) (SoE: IV)

57. It is recommended that the decision whether to perform a radiographic study should be made by a physician specialized in spinal deformities (SoR: A) (SoE: V)

58. It is recommended that frontal radiographic studies are made postero-anteriorly, using digital films with a ratio x-rays, including visualization of the femoral heads and protection of the gonads, in any standing position without the use of support aids or indication of correct posture, unless otherwise justified in the opinion of a clinician specialized in spinal deformities (SoR: A) (SoE: IV)

59. It is recommended that curve magnitude is measured using the Cobb method (SoR: A) (SoE: V)

60. It is recommended that vertebral rotation is measured on the apical vertebra using either the Perdriolle torsiometer or the Raimondi tables/ruler (SoR: B) (SoE: IV)

61. It is recommended that the first and last radiographic evaluation include also a standing lateral view (SoR: A) (SoE: V)

62. On radiographic lateral view, the patient's upper extremities should be placed in a position to uncover the upper thoracic spine. The recommended positions comprise: (1) 45° angle flexion of the arms, elbows extended and hands resting on a support to preserve the sagittal curvature of the spine, (2) the arms crossed over the breasts, (3) the hand resting on the ipsilateral shoulder without pressing it (SoR: B) (SoE: IV)

63. To reduce the invasiveness of follow-up, it is recommended that no more than 1 radiographic study per year should be performed, unless it is truly necessary and is decided by a clinician specialized in spinal diseases (SoR: B) (SoE: IV)

64. To reduce the invasiveness of follow-up, it is recommended that the least number of projections is made on radiographic studies (SoR: A) (SoE: V)

65. It is recommended that all idiopathic scoliosis patients, even if not treated, are regularly followed-up (SoR: A) (SoE: V)

## Conclusions and future research needs

These Guidelines represent a significant improvement when compared to the previous experiences produced either internationally by SOSORT or nationally by other groups [1-4,378]. They have been a big effort of the Commission and the Society to paint the actual situation in this field, starting from the actual evidence, and trying to fill at best all the gray areas not covered by the literature, through the well experimented SOSORT Consensus methodology [38,42,101,114,130,305,329,379].

Like always, Guidelines offers an overview of the evidence in a specific field, and consequently give insights to researchers on which area should be studied more. Looking at Tables 8 and 9, that resume the final grading of the Recommendations in terms of Strength of Evidence and Strength of Recommendations respectively, it is possible to understand the already underlined lack of research in general in this specific area [99,100,260,380]: no evidences of strength level I, very few of level II.

**Table 8:** Strength of Evidence of the approved Recommendations

	I	II	III	IV	V	VI	Total
Bracing	0	0	2	7	8	3	**20**

Specific exercises to prevent scoliosis progression during growth	0	1	2	0	1	4	**8**

Specific exercises during brace treatment and surgical therapy	0	1	1	2	1	0	**5**

Other conservative treatments	0	0	0	0	1	2	**3**

Respiratory function and exercises	0	0	0	2	1	0	**3**

Sports activities	0	0	2	1	2	1	**6**

Assessment	0	0	0	8	12	0	**20**

**Total**	**0**	**2**	**7**	**20**	**26**	**10**	**65**

**Table 9:** Strength of Recommendations

	A	B	C	D	Total
Bracing	4	15	1	0	**20**

Specific exercises to prevent scoliosis progression during growth	0	8	0	0	**8**

Specific exercises during brace treatment and surgical therapy	0	5	0	0	**5**

Other conservative treatments	0	3	0	0	**3**

Respiratory function and exercises	0	3	0	0	**3**

Sports activities	0	4	2	0	**6**

Assessment	9	11	0	0	**20**

**Total**	**13**	**49**	**3**	**0**	**65**

We invite researchers to join this effort, and clinicians to develop good research strategies allowing us the collection of useful data and new evidence.

## Competing interests

All Commission Members are physicians, orthothists and physiotherapists who earn from their own work. The conflict of interests declared by the authors are:

Stefano Negrini has a stock of ISICO (Italian Scientific Spine Institute), Italy

Dimitris Papadopoulos is the major shareholder of Spondylos Laser Spine Lab, Greece.

Manuel Rigo is advisor of Ortholutions, Germany.

Charles H Rivard is consultant to Spinecorporation Ltd, UK.

Michele Romano has a stock of ISICO (Italian Scientific Spine Institute), Italy

Hans-Rudolf Weiss is advisor of Koob-Scolitech, Abtweiler, Germany.

James H. Wynne is an employee of Boston Brace Corp., USA:

No other conflict of interests have been declared.

## Authors' contributions

SN prepared all versions of the document collating all suggestions; proposed, and made the final version of methodology; prepared all versions of flow-charts collating all suggestions. AGA reviewed and approved methodology; revised the initial document; contributed to the development of the clinical practice flow-charts. LA reviewed and approved methodology; revised the initial document; contributed to the development of the clinical practice flow-charts. ABC reviewed and approved methodology; contributed to the development of the clinical practice flow-charts. JCDM reviewed and approved methodology; revised the initial document; contributed to the development of the clinical practice flow-charts. JD revised the initial document; contributed to the development of the clinical practice flow-charts. TBG revised the initial document; contributed to the development of the clinical practice flow-charts. PK approved the flow-charts. TK reviewed and approved methodology; revised the initial document; contributed to the development of the clinical practice flow-charts. TM contributed to the development of the clinical practice flow-charts. SM reviewed and approved methodology; made the final methodological review of the manuscript. JOB made the final practical review of the manuscript. DP reviewed and approved methodology; revised the initial document. MaRi reviewed and approved methodology; revised the initial document; contributed to the development of the clinical practice flow-charts. CHR revised the initial document; contributed to the development of the clinical practice flow-charts. MiRo reviewed and approved methodology; revised the initial document. JHW reviewed and approved methodology; revised the initial document. MV reviewed and approved methodology; revised the initial document. HRW approved the flow-charts. FZ reviewed and approved methodology; revised the initial document; contributed to the development of the clinical practice flow-charts. All authors read and approved the final manuscript.

## Supplementary Material

Additional file 1 **Appendix-Methods**. This file contains a complete and accurate description of the methodology followed during of these Guidelines development. It also includes contributions of the single authors, as well as explicative tables. Click here for file
