# Supplementary material for: 2011 SOSORT guidelines: Orthopaedic and Rehabilitation treatment of idiopathic scoliosis during growth
Source: Scoliosis. 2012 Jan 20;7:3. doi: 10.1186/1748-7161-7-3 (PMC3292965; doi:10.1186/1748-7161-7-3)
Supplement: Additional file 1 — Appendix-Methods. This file contains a complete and accurate description of the methodology followed during of these Guidelines development. It also includes contributions of the single authors, as well as explicative tables. [file 1748-7161-7-3-S1.DOC]

# Appendix. Methods

### Methodology of Guidelines development

#### Review of previous Guidelines

Work started through a careful research of the literature to find previous versions of the “Guidelines on Scoliosis Conservative Treatment.” These have been searched through a comprehensive bibliographic search on Medline with the key word “Scoliosis” and “Guidelines”. We found the following:

- SOSORT (2006) [1]: these have the advantage of giving immediate choices following diagnostic therapeutic pathways, but the limitation of not being methodologically well developed.
- Italian (2005) [2]: these have the advantage of being methodologically well developed, but too lengthy and detailed, and organized through recommendations and not immediate therapeutic pathways like the SOSORT guidelines.
- French (2008) [3]: generic and descriptive in nature.
- Polish (2009) [4]: direct translation of the SOSORT Guidelines.

Each single Guideline has been evaluated according to the AGREE checklist (i.e. to the actual recognised standard for Guidelines production) by two members of the Commission (coordinator, and methodologist) [378] (Additional File 1, Table S1) with the aim or verifying their main strengths and limits. The following SOSORT members also participated in this review: Negrini S, Minozzi S and Zaina F.

The previous Guidelines, from which considerable input came, had been authored by the following SOSORT members: Aulisa L, Czaprowski D, Durmala J, Fraschini P, Grivas T, Kowalski IM, Kotwicki T, Landauer F, Maruyama T, Negrini S, Rigo M, Simonazzi P, Weiss HR. The following non-SOSORT members were included: Ferraro C, Glowacki M, Hawes M, Kolban M, Masiero S, Sliwinski Z, Snela S, Tedeschi C, Venturin A.

A document merging from all the previous Guidelines the pertinent and most methodologically sound parts in a unique and uniform text has been prepared by the Coordinator.

#### Stage 1: Approval of methodology

In the first round of votes, the methodology proposed by the SOSORT Board (here reported in its final version) was approved by Commission members with only slight changes. Moreover, each member of the Commission was asked to approve or propose changes to the “Strength of evidence” table (slightly modified from the previous Italian Guidelines) (Additional File 1, Table S2).

Members who participated in this stage: Aulisa A, Aulisa L, Circo A, De Mauroy JC, Kotwicki T, Minozzi S, Negrini S, Papadoupoulus D, Parent E, Rigo M, Romano M, Wynne J, Villagrasa M, Zaina F.

#### Stage 2: Refining the initial document

The initial document was divided into four sections and each member of the Commission worked directly on one of them in a way to have at least two people per section (in the end, we had a range of 3 to 5 versions per part). The sections were:

1. Introduction
2. Assessment
3. Specific exercises, respiratory exercises, specific exercises during brace and surgical treatment, sport activities
4. Brace treatment

The following tasks were then performed by the members of the commission in each section:

1. Completion of the literature search
2. Proposal for the revision of the literature review
3. Vote and suggestions for change of the existing recommendations
4. Development of new recommendations

Members who participated in this stage: Aulisa A, Aulisa L, De Mauroy JC, Durmala J, Grivas TB, Kotwicki T, Negrini S, Papadoupoulus D, Rigo M, Rivard C, Romano M, Wynne J, Villagrasa M, Zaina F.

#### Stage 3: Development of clinical practice flow-charts

To prepare the Practical Approach section of these Guidelines, a table that included each member of the Commissions presentation of his/her own evidence based clinical practice pathways according to the different classifications of scoliosis was prepared (Additional File 1, Table S3). Due to the needed clinical and professional background, this table was completed only by participating physicians. Following the usual practice in the literature for all these kinds of schemes [255, 256], it was based on Cobb degrees, type of scoliosis, and bone age. The weakness of this model is recognised and limitations were carefully listed by participants to the Commission to be reported in the final document. To facilitate the voting process, a table that included the previous SOSORT Guidelines treatment scheme was added (Additional File 1, Table S4).

During the SOSORT Board Meeting that gave raise to the New Guidelines project, it was decided not to follow the Lonstein and Carlson progression formula as the guide of clinical decisions for AIS as proposed in the Old SOSORT Guidelines, since this model has been widely criticised. It was also decided that the Lonstein and Carlson formula, as well as the other predictors of scoliosis progression would be listed in the final document.

In the same table, the goals of treatment for each different clinical situation was voted on as well (Additional File 1, Table S4).

Members who participated in this stage: Aulisa A, Aulisa L, Circo A, De Mauroy JC, Durmala J, Grivas TB, Kotwicki T, Negrini S, Rigo M, Rivard C, Zaina F.

#### Stage 4: Second vote on the clinical practice flow-charts

All votes coming from the “Stage 3” procedure were considered by the Coordinator. First, a table grading the “strength” of the treatment proposed was prepared (Additional File 1, Table S5); then, in front of each single clinical situation was listed the range of treatments that could be possibly proposed using to a fully evidence-based clinical practice approach. This scheme was based on the previous one (Additional File 1, Table S4), and included a range from a minimum to a maximum of intensity of treatment for each single clinical situation (Additional File 1, Table S5). This was then voted on point by point, stating for each single cell if the member:

- fully agreed
- did not agree totally, but could accept
- could not accept

Suggestions were required as well.

Members who participated in this stage: Aulisa A, Aulisa L, Circo A, De Mauroy JC, Durmala J, Grivas TB, Kotwicki T, Negrini S, Rigo M, Rivard C, Zaina F.

#### Stage 5: Final votes on the clinical practice flow-charts

All votes coming from the “Stage 4” procedures were considered by the Coordinator. Based on the suggestions that were received, the last review of the table grading the “strength” of the treatment was prepared and approved. The clinical situations in which all reviewers have given either a total agreement, or a an acceptability vote was accepted (36.3%). Since some uncertainty remained (Additional File 1, Table S6), a new proposal that incorporated the received suggestions was prepared and sent out. During these final revisions, the following instructions were given to the reviewers:

- You DO NOT have to mark what you don't do. You have to mark ONLY what you would consider COMPLETELY WRONG if made by a colleague, because you think it is ABSOLUTELY over-treatment. Mark the cell in black, so that all these points will be discussed in Barcelona if at least two of us will mark it as ABSOLUTELY INCORRECT. If what you would do is a treatment less strong than what is written, but you think that what is written could be done by others without being TOTALLY WRONG, don't mark the cell.
- You DO NOT have to mark what you don't do. You have to mark ONLY what you would consider COMPLETELY WRONG if made by a colleague, because you think it is ABSOLUTELY under-treatment. Mark the cell you consider wrong in black, so that all these points will be discussed in Barcelona if at least two of us will mark it as ABSOLUTELY INCORRECT. If what you would do is a treatment more strong than what is written, but you think that what is written could be done by others without being TOTALLY WRONG, don't mark the cell.

In this way we asked only for what was considered totally unacceptable because that specific “maximum” intensity of treatment was considered by the voter as an “over-treatment”, or vice versa that specific “minimum” intensity of treatment was considered by the voter as an “under-treatment”. All points that were marked by at least one of the reviewers was noted to be discussed during the Consensus Session in Barcelona.

Members who participated in this stage: De Mauroy JC, Grivas TB, Knott P, Maruyama T, Negrini S, Weiss HR, Zaina F.

#### Stage 6: Final revision of the last document

All suggestions coming from all previous stages were included in the last version of the 2011 SOSORT Guidelines on “Idiopathic Scoliosis Conservative Treatment during growth”. At this stage the last document was split in four Parts to make each of them easy to read. In preparation of the last document the coordinator performed another final complete literature review reported in the Methods section of each Part. The final text included the complete review, all suggestions received, and proposals to reach agreement on the discordances previously reported by the Commission.

Each single Part of the last document was sent to all Commission and SOSORT Board members. After receiving their final suggestions, the documents were re-arranged into three parts and sent out to all SOSORT members to be read before the final general discussion during the 8^th^ SOSORT Meeting in Barcelona.

#### Methodological and practical review

The last documents were sent also to the methodologist and the patient participating in the Commission, so that their suggestions could be included in the final document.

Members who participated in this stage: Minozzi S, and O’Brien J.

#### Review by the SOSORT Members

After review according to “Stage 6” the final documents with all “points to be discussed” were sent to all SOSORT Members to collect their comments and suggested changes before the SOSORT Meeting.

#### Consensus Session during the SOSORT Meeting

According to the Methodology used during the production of the previous SOSORT Consensus Guidelines [4, 38, 42, 101, 114, 130, 213, 305, 329, 377, 379], during the Consensus Session held in May 20^th^ 2011 at the 8^th^ SOSORT Meeting in Barcelona, all Recommendations were individually reviewed and approved.

The discussion points raised during the Stage 6 procedure on the final documents were presented and discussed to be incorporated in the final document, and the consequent changes immediately proposed and discussed. The Strength of Evidence levels V and VI were obtained according to the votes of participants (90% of agreement, or 70-89% respectively), and all Recommendations that did not reach at least 70% of agreement were rejected.

At this stage a Strength of Recommendation (SoR) scale was adopted (Additional File 1, Table S7) that shows the importance each Recommendation should have in the clinical everyday world, balancing all typical factors involved in this decision (patients, professionals, social). The Strength of Recommendation scale is meant to accompany and complement the Strength of Evidence scale. Participants were asked to vote and the lowest grade that received at least 80% of the total votes was selected as the final one (e.g. in the case of the following votes: A=60%, B=10%, C=20%, and D=10% the final SoR was a C; in the case of the following votes: A=20%, B=60%, C=15%, and D=5%, the final SoR was a B).

The SOSORT Members who participated in the Consensus Session and gave their final votes and contributions were: Aulisa Angelo, Aulisa Lorenzo, Barral Frederic, Betts Tony, Chowanska Joanna, Cohen Larry, Czaprowski Dariusz, De Mauroy Jean Claude, Durmala Jacek, Gil Josè Maria, Gloria Salvà, Grivas Theodor B., Hermus Joris, Janssen Beth, Kaced Hauria, Knott Patrick, Kotwicki Tomasz, Kowalski Ireneusz, Lebel Andrea, Martin Matthews, Maruyama Toru, Maude Erika, Maude Jane, M'hango Andrej, Moramarco Marc, Negrini Stefano, Neuhaus Sulam, Neuhory Tamar, Papadopoulos Dimitris, Raice Ester, Rajter Hagit, Rigo Manuel, Rivett Louann, Selvadurai, Shnier Gavin, Simonazzi Paolo, Tessadri Fabrizio, Torres Beatriz, Wynne James.

Other attenders of the meeting giving their contribution, and their names and/or nicknames, were :

Akgay Burgin, Berkowitz Rowan, Black D.A. Jason, Chekryzhev non è a posto con i pagamenti, Chungwai Chou, Closee, Corbella C., Courtis Isabelle, Dorman Rebecca, Escalada Ferran, Floch Sabine, Hobson Kylie, Laita Judith, Lopez Ana, Plaszewski Maciej, Reugina Liza, Sanja, Tueba Kuru, Wellburn Shaun, Wentzy P, Wilsom Amy.

The General results of the SOSORT Consensus Session is reported in Additional File 1, Table S8.

# Tables

Table S1. Strength of Evidence grading used in these Guidelines. Questions on Effectiveness (treatment results) and Diagnosis (assessment) have been considered

| Strength of evidence | Question | **Meaning** |
| --- | --- | --- |
| I | Effectiveness | Multiple Randomized Controlled Trials or Systematic Reviews of such studies |
|  | Diagnosis | Multiple Randomized Controlled Trials, or Cross-sectional Studies with verification by reference (gold) standard, or Systematic Reviews of such studies |
| **II** | Effectiveness | One Randomized Controlled Trial |
|  | Diagnosis | One Randomized Controlled Trial, or one Cross-sectional Study with verification by reference (gold) standard |
| **III** | Effectiveness | Multiple Controlled nonrandomized Studies or Systematic Reviews of such studies |
|  | Diagnosis | Multiple Cross-sectional Studies with incomplete & unbalanced verification with reference (gold) standard |
| **IV** | Effectiveness | Other studies |
|  | Diagnosis |  |
| **V** | Effectiveness | SOSORT Consensus with more than 90% of agreement |
|  | Diagnosis |  |
| **VI** | Effectiveness | SOSORT Consensus with 70 to 89% of agreement |
|  | Diagnosis |  |

Table S2. Quality of the previously published Guidelines on Conservative treatment of scoliosis according to the AGREE instrument [378]

| **AGREE Instrument** | | **Guidelines** | | | |
| --- | --- | --- | --- | --- | --- |
| **Domain** | **Items** | **SOSORT [1]**  **(2006)** | **Italian [2]**  **(2005)** | **French [3]**  **(2008)** | **Polish [4]**  **(2009)** |
| Scope and Purpose | 3 | 2.0 | 3.0 | 3.7 | 2.0 |
| Stakeholder involvement | 4 | 1.3 | 2.5 | 2.3 | 1.3 |
| Rigour of development | 7 | 1.0 | 3.3 | 1.1 | 1.1 |
| Clarity and presentation | 4 | 2.5 | 2.0 | 2.5 | 2.5 |
| Applicability | 3 | 1.0 | 1.0 | 1.0 | 1.0 |
| Editorial indipendence | 2 | 2.5 | 2.5 | 1.0 | 2.5 |
| Total | 23 | 1.6 | 2.5 | 1.9 | 1.6 |

Table S3. Scheme for voting on the evidence-based clinical practice approach to scoliosis by each MD participating in the Commission

|  |  | **Your vote on therapeuthic decisions** | | | | | | | | |
| --- | --- | --- | --- | --- | --- | --- | --- | --- | --- | --- |
|  |  | Cobb degrees | | | | | | | | |
|  | Modifiers | 0-10 | 11-15 | 16-20 | 21-25 | 26-30 | 31-35 | 36-40 | 41-45 | 46-50 |
| Infantile |  |  |  |  |  |  |  |  |  |  |
| Juvenile |  |  |  |  |  |  |  |  |  |  |
| Adolescent | Risser 0 |  |  |  |  |  |  |  |  |  |
|  | Risser 1 |  |  |  |  |  |  |  |  |  |
|  | Risser 2 |  |  |  |  |  |  |  |  |  |
|  | Risser 3 |  |  |  |  |  |  |  |  |  |
|  | Risser 4 |  |  |  |  |  |  |  |  |  |
|  | Risser 4-5 |  |  |  |  |  |  |  |  |  |
| Adult | No pain |  |  |  |  |  |  |  |  |  |
|  | Chronic Pain |  |  |  |  |  |  |  |  |  |
| Elderly | No pain |  |  |  |  |  |  |  |  |  |
|  | Chronic Pain |  |  |  |  |  |  |  |  |  |
|  | Decompensation |  |  |  |  |  |  |  |  |  |
|  |  |  |  |  |  |  |  |  |  |  |
| Obeservation | Ob+months |  | **Your vote on aim of treatment** | | | | | | | |
| Specific Exercises | Ex |  | Aim | | Cobb degrees | | | | | |
| Inpatient rehabilitation | SIR |  |  |  | 11-30 | 31-35 | 36-45 | Over 45 | Juvenile | Infantile |
| Soft bracing | SB |  | Absolute | | Avoiding surgery | | | | | |
| Rigid bracing | RB+hours |  | Primary | | 20-25 | | 10-34 | | | |
| Surgery | Su |  | Secondary | | 10-19 | No progression | | | 35-45 | |

Table S4. The example of a possible vote, used to facilitate the voting process, obtained during the previous SOSORT Guidelines treatment scheme

|  |  | **Previous SOSORT Guidelines** | | | | | | | | |
| --- | --- | --- | --- | --- | --- | --- | --- | --- | --- | --- |
|  |  | Cobb degrees | | | | | | | | |
|  | Modifiers | 0-10 | 11-15 | 16-20 | 21-25 | 26-30 | 31-35 | 36-40 | 41-45 | 46-50 |
| Infantile |  |  | Ob 6-12 | Ex | | Br 12-16 | | | | |
| Juvenile |  |
| Adolescent | Risser 0 | See below | | | | | | | | |
|  | Risser 1 |
|  | Risser 2 |
|  | Risser 3 |
|  | Risser 4 |  | Obs 6-12 | | Ex | | | Br 16 | | |
|  | Risser 4-5 |  | | | | Ex | | Br 16 for aesthetics | | |
| Adult | No pain |  | | | | | Ex | | | |
|  | Chronic Pain |  | Ex | | | | | | | |
| Elderly | No pain |  | | | | | | | | |
|  | Chronic Pain |
|  | Decompensation |
|  |  | Progression Factor Lonstein Carlson | | | |  |  |  |  |  |
|  |  | 0-40% | 40% | 60% | 80% |  |  |  |  |  |
| Adolescent | Risser 0-3 | Obs 3 | Ex | Br 16 | Br 23 |  |  |  |  |  |
|  |  |  |  |  |  |  |  |  |  |  |
| Obeservation | Ob+months |  |  |  |  |  |  |  |  |  |
| Specific Exercises | Ex |  |  |  |  |  |  |  |  |  |
| Inpatient rehabilitation | SIR |  |  |  |  |  |  |  |  |  |
| Surgery | Su |  |  |  |  |  |  |  |  |  |

Table S5. Final proposal of a possible clinical approach to scoliosis obtained from the previous votes, and sent out for another round. Below, the proposed table of the “Strength” of treatment used for idiopathic scoliosis can be found, as well as the resuming “goal of treatment” table

|  |  |  | Cobb degrees | | | | | | | | | |
| --- | --- | --- | --- | --- | --- | --- | --- | --- | --- | --- | --- | --- |
|  | Modifiers | Range indication | 0-10 | 11-15 | 16-20 | 21-25 | 26-30 | 31-35 | 36-40 | 41-45 | 46-50 | >50 |
|  |  |  |  |  |  |  |  |  |  |  |  |  |
| Infantile |  | Min | Obs 3 mo | Obs 3 mo | Obs 3 mo | SB | SB | SB | SB | SB | SB | FTRB |
|  |  | Max | Obs 6 mo | Obs 6 mo | SB | FTRB | FTRB | FTRB | FTRB | FTRB | Su | Su |
|  |  |  |  |  |  |  |  |  |  |  |  |  |
| Juvenile |  | Min | Obs 6 mo | Obs 6 mo | Obs 6 mo | SB | SB | SB | SB | SB | SB | FTRB |
|  |  | Max | Obs 8 mo | Ex | SB | FTRB | FTRB | FTRB | FTRB | FTRB | Su | Su |
|  |  |  |  |  |  |  |  |  |  |  |  |  |
| Adolescent | Risser 0 | Min | Obs 3 mo | Obs 6 mo | Obs 6 mo | Ex | SB | SB | SB | SB | SB | FTRB |
|  |  | Max | Obs 6 mo | Ex | SB | FTRB | FTRB | FTRB | FTRB | FTRB | Su | Su |
|  |  |  |  |  |  |  |  |  |  |  |  |  |
|  | Risser 1 | Min | Obs 3 mo | Obs 6 mo | Obs 6 mo | Ex | SB | SB | SB | SB | SB | FTRB |
|  |  | Max | Obs 6 mo | Ex | SB | FTRB | FTRB | FTRB | FTRB | FTRB | Su | Su |
|  |  |  |  |  |  |  |  |  |  |  |  |  |
|  | Risser 2 | Min | Obs 6 mo | Obs 6 mo | Obs 6 mo | Ex | SB | SB | SB | SB | SB | FTRB |
|  |  | Max | Obs 8 mo | Ex | SB | FTRB | FTRB | FTRB | FTRB | FTRB | Su | Su |
|  |  |  |  |  |  |  |  |  |  |  |  |  |
|  | Risser 3 | Min | Obs 6 mo | Obs 6 mo | Obs 6 mo | Obs 6 mo | Ex | SB | SB | SB | SB | FTRB |
|  |  | Max | Obs 8 mo | Ex | PTRB | FTRB | FTRB | FTRB | FTRB | FTRB | Su | Su |
|  |  |  |  |  |  |  |  |  |  |  |  |  |
|  | Risser 4 | Min |  | Obs 6 mo | Obs 6 mo | Obs 6 mo | Obs 6 mo | Obs 6 mo | Obs 6 mo | Obs 6 mo | SB | FTRB |
|  |  | Max | Obs 8 mo | Ex | PTRB | FTRB | FTRB | FTRB | FTRB | FTRB | Su | Su |
|  |  |  |  |  |  |  |  |  |  |  |  |  |
|  | Risser 4-5 | Min |  | Obs 6 mo | Obs 6 mo | Obs 6 mo | Obs 6 mo | Obs 6 mo | Obs 6 mo | Obs 6 mo | SB | FTRB |
|  |  | Max | Obs 12 mo | Ex | PTRB | FTRB | FTRB | FTRB | FTRB | FTRB | Su | Su |
|  |  |  |  |  |  |  |  |  |  |  |  |  |
| Adult | No pain | Min | Nothing | Nothing | Nothing | Obs 12 mo | Obs 12 mo | Obs 12 mo | Obs 12 mo | Obs 12 mo | Obs 12 mo | Obs 12 mo |
|  |  | Max | Obs 12 mo | Obs 12 mo | Obs 12 mo | Obs 36 mo | Obs 36 mo | Obs 36 mo | Obs 12 mo | Obs 12 mo | Obs 12 mo | Obs 12 mo |
|  |  |  |  |  |  |  |  |  |  |  |  |  |
|  | Chronic Pain | Min | Nothing | Ex | Nothing | Ex | Ex | Ex | Ex | Ex | Ex | Ex |
|  |  | Max | SB | SB | SB | SB | SB | Su | Su | Su | Su | Su |
|  |  |  |  |  |  |  |  |  |  |  |  |  |
| Elderly | No pain | Min | Nothing | Nothing | Nothing | Nothing | Obs 12 mo | Obs 12 mo | Obs 12 mo | Obs 12 mo | Obs 12 mo | Obs 12 mo |
|  |  | Max | Obs 12 mo | Obs 12 mo | PTRB | Obs 12 mo | Obs 12 mo | Obs 12 mo | Obs 12 mo | Obs 12 mo | Obs 12 mo | Obs 12 mo |
|  |  |  |  |  |  |  |  |  |  |  |  |  |
|  | Chronic Pain | Min | Nothing | Ex | Nothing | Ex | Ex | Ex | Ex | Ex | Ex | Ex |
|  |  | Max | SB | SB | PTRB | SB | SB | SB | SB | PTRB | PTRB | PTRB |
|  |  |  |  |  |  |  |  |  |  |  |  |  |
|  | Decompensation | Min | Nothing | Nothing | Nothing | Ex | Ex | Ex | Ex | Ex | Ex | Ex |
|  |  | Max | PTRB | PTRB | PTRB | PTRB | PTRB | PTRB | PTRB | PTRB | PTRB | PTRB |
|  |  |  |  |  |  |  |  |  |  |  |  |  |
|  |  | | Min | | --- | |  |  |  |  |  |  |  |  |  |  |
| Nothing | Nothing |  | **Goal of treatment** | | | | | | | | |  |
| Observation | Ob+months |  | Aim | |  | Cobb degrees | | | | | |  |
| Specific Exercises | Ex |  |  |  |  | 11-30 | 31-35 | 36-45 | Over 45 | Juvenile | Infantile |  |
| Inpatient rehabilitation | SIR |  | Absolute | |  | Avoid surgery | | | | | |  |
| Night-time Rigid Bracing (8-12 hours) | NTRB |  | Primary | | Min | 10 | | | | | |  |
| Soft bracing | SB |  |  |  | Max | 25 | | 34 | | | |  |
| Part-Time Rigid Bracing (12-20 hours) | PTRB |  | Secondary | | Min | 10 | | | No progression | | |  |
| Full-time Rigid bracing (20-24 hours) | FTRB |  |  |  | Max | 30 | | | No progr. | 45 | |  |
| Surgery | Su |  |  |  |  |  |  |  |  |  |  |  |
|  |  | Max |  |  |  |  |  |  |  |  |  |  |

Table S6. Results of the Stage 4 votes on each clinical situation

| **Results of votes** | **Clinical situations voted** | **%** |
| --- | --- | --- |
| Approved | 111 | 36.3% |
| Not approved but not changed | 113 | 36.9% |
| Not approved and changed | 57 | 18.6% |
| Rejected and to be discussed | 25 | 8.2% |
| **Total** | **306** |  |

Table S7. Strength of Recommendations grading used in these Guidelines.

| **Strength of recommendation** | **Meaning** |
| --- | --- |
| **A** | it must be applied widely and to all patients with this specific need |
| **B** | it is important, but can be applied not to all patients with this specific need |
| **C** | less important, it can be applied on a voluntary basis |
| **D** | very low importance |

Table S8. Results of the Consensus Session held in Barcelona during the 8^th^ SOSORT Meeting

| **Results of votes on Recommendations** | **Clinical situations voted** | **%** |
| --- | --- | --- |
| Approved by 90%  (Consensus V if not already defined by the literature) | 34 | 46.6 |
| Approved by 70-89.9%  (Consensus VI if not already defined by the literature) | 31 | 42.5 |
| Rejected | 8 | 10.9 |
| **Total** | **73** |  |
